# Oncolytic virus-mediated p53 activation boosts the antitumor immunity of a p53-transduced dendritic cell vaccine

**DOI:** 10.1038/s41541-025-01219-5

**Published:** 2025-07-19

**Authors:** Motohiko Yamada, Hiroshi Tazawa, Kanto Suemori, Naohiro Okada, Yoshinori Kajiwara, Ryohei Shoji, Yasuo Nagai, Hiroaki Inoue, Naoyuki Hashimoto, Nobuhiko Kanaya, Satoru Kikuchi, Shinji Kuroda, Hiroyuki Michiue, Yasuo Urata, Shunsuke Kagawa, Toshiyoshi Fujiwara

**Affiliations:** 1 Department of Gastroenterological Surgery, Okayama University Graduate School of Medicine, Dentistry and Pharmaceutical Sciences, Okayama, 700-8558 Japan; 2 Center for Innovative Clinical Medicine, Okayama University Hospital, Okayama, 700-8558 Japan; 3 Neutron Therapy Research Center, Okayama University Hospital, Okayama, 700-8558 Japan; 4 Oncolys BioPharma, Inc, Tokyo, 105-0001 Japan

**Keywords:** Colorectal cancer, Cancer immunotherapy

## Abstract

Dendritic cells (DCs) transduced with replication-deficient, wild-type human p53-expressing adenovirus Ad-p53 (Ad-p53 DCs) induce p53-targeting cytotoxic T lymphocytes (CTLs). However, the antitumor efficacy of Ad-p53 DCs is diminished by weak p53 immunogenicity in tumor cells and poor immune responses. We developed a p53-armed oncolytic adenovirus, OBP-702, to induce tumor-specific p53 expression and antitumor immune response, suggesting a role for OBP-702 in enhancing the antitumor efficacy of Ad-p53 DCs. The combined effect of Ad-p53 DCs and OBP-702 was investigated using murine colon cancer (CC) tumor models. Ad-p53 DCs were obtained by stimulating bone marrow-derived cells with granulocyte-macrophage colony-stimulating factor, interleukin-4, and Ad-p53. Subcutaneous tumor models of CT26 (p53 wild-type) and MC38 (p53 mutant-type) murine CC cell lines were used to evaluate the therapeutic potential of combination therapy in the terms of tumor growth, abscopal effect, antitumor immune response, and presentation of p53 peptides in tumor cells. Combination therapy with Ad-p53 DCs and OBP-702 significantly suppressed the growth of p53-intact CT26 tumors at treated and untreated sites by inducing tumor-infiltration of CD8+ CTLs and CD11c+ DCs. OBP-702-infected tumor cells presented human p53 epitopes in the context of major histocompatibility complex molecules, which were recognized by CTLs induced by Ad-p53 DCs. Combination therapy significantly suppressed the growth of p53-mutant MC38 tumors by activating the antitumor immune response. Our results suggest that OBP-702-mediated presentation of p53 epitopes on tumor cells enhances the antitumor efficacy of Ad-p53 DCs against murine CC tumors by attracting p53-targeting CTLs.

## Introduction

Dendritic cell (DC)-based vaccine therapy is a type of immunotherapy that induces tumor-associated antigen (TAA)-specific cytotoxic T lymphocytes (CTLs) using TAA-presenting DCs^1^. The tumor suppressor gene *p53* is one of the most frequently mutated genes, and mutant p53 protein is often overexpressed in various cancers^2^. Mutant or wild-type p53 epitopes derived from p53 protein are presented as TAAs in the context of major histocompatibility complex (MHC) molecules on cancer cells^3^. Although mutant p53 neoepitopes are immunogenic as TAAs, CTLs targeting mutant p53 neoepitopes are applicable for only small populations of cancers harboring identical mutations in the *p53* gene^4^. Therefore, wild-type p53 epitopes are used as TAAs more frequently than mutant p53 neoepitopes because most tumor cells expressing mutant p53 proteins can be recognized by CTLs targeting wild-type p53 epitopes^3,4^.

Several DC-based vaccine therapies targeting wild-type p53 epitopes have been developed to induce p53-targeting CTLs^5^. For instance, clinical studies have examined using DCs transduced with wild-type, p53-expressing, replication-deficient adenovirus Ad-p53 (Ad-p53 DCs) or modified vaccinia Ankara virus p53MVA (p53MVA DCs) to treat cancer patients in combination with chemotherapy^5^. Immunization with Ad-p53 DCs or p53MVA DCs was shown to induce p53-specific CTLs in preclinical and clinical studies^5^. However, the antitumor efficacy of wild-type p53-targeting DC vaccine therapy is limited due to weak p53 immunogenicity in tumor cells, poor tumor-infiltration of CTLs, and formation of an immunosuppressive tumor microenvironment (TME). Therefore, the development of novel antitumor modalities to activate p53 immunogenicity in tumor cells and promote tumor-infiltration of CTLs are needed to improve the antitumor activity of p53-targeting DC vaccine therapy.

Oncolytic virotherapy has emerged as a novel antitumor modality in combination with chemotherapy, radiotherapy, and immunotherapy^6,7^. We developed a telomerase-specific, replication-competent oncolytic adenovirus, OBP-301 (suratadenoturev)^8^. OBP-301 exhibits broad-spectrum antitumor activity against malignant tumor cells with telomerase activity^9^. OBP-301 has the therapeutic potential to enhance the sensitivity of cancer cells to chemotherapy^10^, radiotherapy^11^, and immune checkpoint inhibitors (ICIs)^12^. To enhance the therapeutic potential of OBP-301, we generated a p53-armed virus, OBP-702, that induces the tumor suppressor gene *p53*^13^. OBP-702 exhibits stronger antitumor activity than non-armed OBP-301 via activation of the p53 signaling pathway and induction of apoptosis and autophagy of tumor cells^14^. OBP-702-mediated p53 overexpression contributes to the induction of immunogenic cell death in murine tumor cells, leading to the tumor-infiltration of CTLs and enhancement of the antitumor efficacy of ICIs^15^. Moreover, OBP-702 was shown to suppress tumor-infiltration of immunosuppressive myeloid-derived suppressor cells in chemo-resistant murine pancreatic cancer^16^. These findings suggest that OBP-702 has the therapeutic potential to activate p53 expression in tumor cells and promote tumor-infiltration of CTLs. Therefore, we hypothesized that combination treatment with OBP-702 would improve the antitumor efficacy of Ad-p53 DC vaccine therapy by activating p53 immunogenicity in tumor cells and tumor-infiltration of CTLs, resulting in enhancement of the antitumor immune response.

In the present study, we investigated the combined effect of Ad-p53 DCs and OBP-702 using murine colon cancer (CC) tumor models utilizing CT26 (wild-type p53) and MC38 (mutant-type p53) cells. The expression levels of DC maturation markers in Ad-p53 DCs were analyzed by flow cytometry. The therapeutic potential and modulation of the TME in combination therapy were analyzed using unilateral and bilateral subcutaneous CC tumor models. An immunopeptidomics approach was used to identify p53 epitopes presented in the context of MHC molecules on Ad-p53 DCs and OBP-702-treated tumor cells. The role of p53 epitopes on OBP-702-treated tumor cells was evaluated by analyzing p53 epitope-responsive CTLs.

## Results

### Characterization of Ad-p53 DCs

**Fig. 1:**
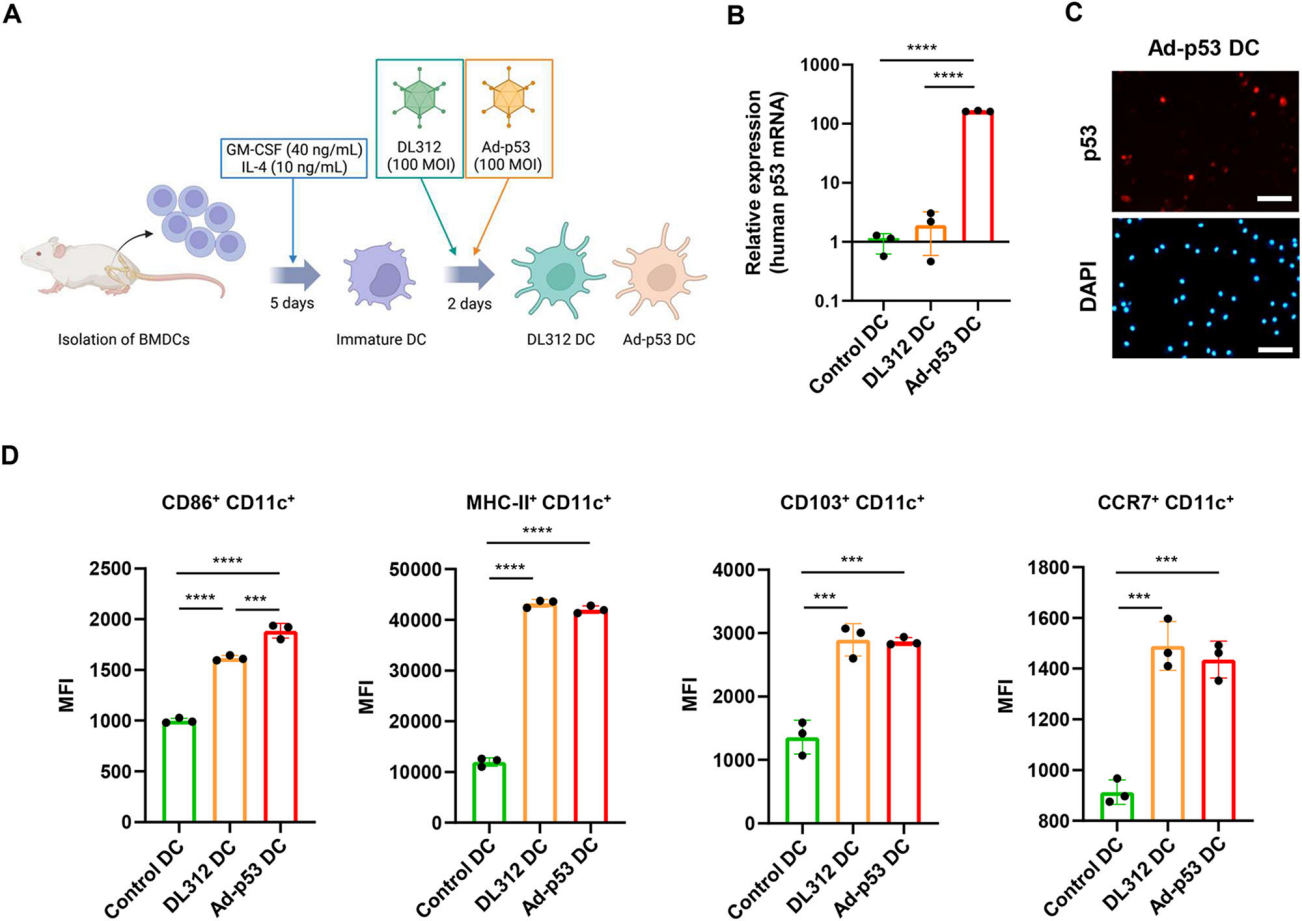
Expression levels of p53 and mature DC markers in Ad-p53 DCs. **A** Schematic illustration of the experimental protocol of preparation of dendritic cells (DCs) transduced with the replication-deficient, wild-type p53-expressing adenovirus Ad-p53 (Ad-p53 DCs). Bone marrow-derived cells isolated from the femur of BALB/c mice were incubated with GM-CSF (40 ng/mL) and IL-4 (10 ng/mL) for 5 days and then infected with Ad-p53 (100 MOI) for 2 days to obtain Ad-p53 DCs. Figures were generated using BioRender. **B** Expression of human p53 mRNA in control DCs and DCs infected with DL312 (100 MOI) or Ad-p53 (100 MOI) for 48 h. GAPDH was used as a control gene. **C** Representative photographs of expression of human p53 protein in DCs infected with Ad-p53 (100 MOI) for 72 h. Scale bars, 100 μm. **D** Expression of mature DC markers in Ad-p53 DCs. Control DCs and Ad-p53 DCs were subjected to flow cytometry for analysis of CD11c, CD86, MHC class-II (MHC-II), CD103, and CCR7 expression. The mean fluorescence intensity (MFI) for each protein was determined by calculating the difference between the MFI of the antibody- and control IgG-treated cells. Data are expressed as mean ± SD (*n* = 3). Statistical significance was determined using one-way ANOVA followed by Tukey’s comparison test. ***P < 0.001; ****P < 0.0001.

To obtain Ad-p53 DCs, BMDCs isolated from the femurs of C57BL/6 J mice were incubated with GM-CSF and IL-4 for 5 days and then infected with replication-deficient Ad-p53 (Supplementary Fig. 1A) for 2 days (Fig. 1A). The replication-deficient *E1A*-deleted adenovirus DL312 (Supplementary Fig. 1B) was used to obtain DL312 DCs as a control for Ad-p53 DCs. Real-time PCR analysis demonstrated that the expression of human p53 mRNA was significantly increased in Ad-p53 DCs compared with control DCs or DL312 DCs, when GAPDH and β-actin were used as control genes (Fig. 1B and Supplementary Fig. 2). Immunocytochemistry analysis showed the presence of human p53 protein in Ad-p53 DCs (Fig. 1C). To evaluate the DC maturation level of Ad-p53 DCs, flow cytometry was used to analyze the expression levels of various DC maturation markers (CD86, MHC-II, CD103) and a DC migration marker (CCR7) in CD11c+ DCs (Supplementary Fig. 3). Ad-p53 DCs and DL312 DCs exhibited a significantly higher proportion of CD11c+ DCs expressing CD86, MHC-II, CD103, and CCR7 compared with control DCs (Fig. 1D). These results suggest that Ad-p53 DCs include mature DCs expressing human p53 protein.

DCs play a crucial role in presenting TAAs to T cells in regional lymph nodes (LNs)^17^. To evaluate the migration of virus-transduced DCs to regional LNs, DCs infected with the green fluorescent protein (GFP)-expressing adenovirus Ad-GFP (Ad-GFP DCs) were injected into the left foot pad of mice, and the left inguinal LN was harvested 24 h after inoculation. Mice injected with Ad-GFP DCs exhibited GFP expression in the regional LNs (Supplementary Fig. 4). These results suggest that adenovirus-infected DCs can migrate to regional LNs.

### In vivo antitumor effect of combination therapy with Ad-p53 DCs and OBP-702 against p53-intact CT26 tumors

**Fig. 2:**
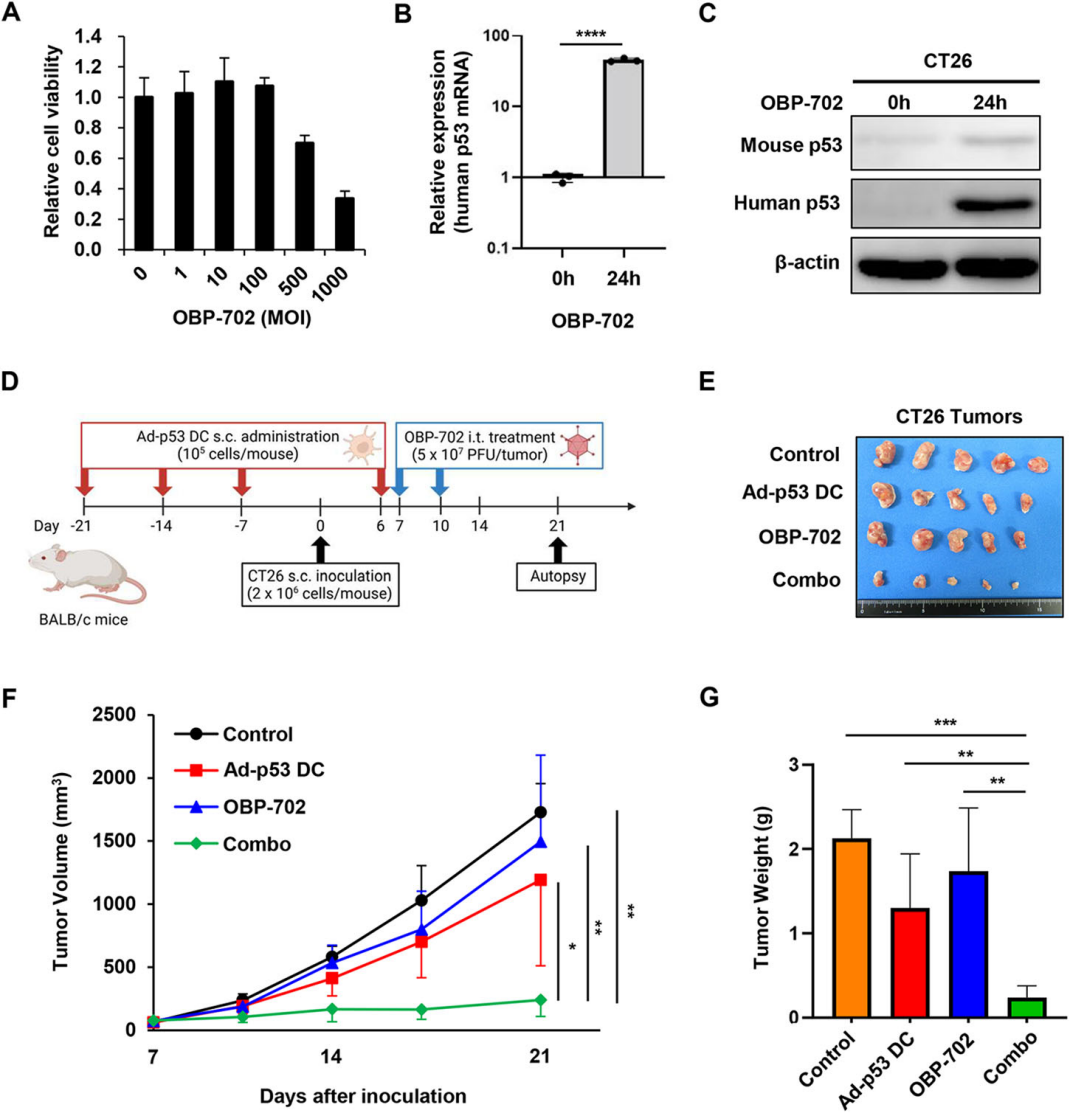
Combined effect of Ad-p53 DCs and OBP-702 in a p53-intact murine CT26 tumor model. **A** Viability of CT26 cells was assessed using an XTT assay 3 days after OBP-702 treatment at the indicated MOIs. Cell viability was calculated relative to that of mock-infected cells, which were set as 1.0. **B** Expression of human p53 mRNA in CT26 cells before and after infection with OBP-702 (100 MOI) for 24 h. Data are expressed as mean ± SD (*n* = 3). Statistical significance was determined using the Student *t*-test. ****P < 0.0001. **C** Whole-cell lysates of CT26 cells before and after infection with OBP-702 (100 MOI) for 24 h were subjected to Western blot analysis of mouse p53, human p53, and β-actin expression. **D** Schematic illustration of the CT26 tumor model experimental protocol. Figures were generated using BioRender. **E** Photographs of tumors in each group. **F** Growth curves for control, Ad-p53 DC-treated, OBP-702-treated, and combination therapy (combo)-treated tumors. **G** Weight of control, Ad-p53 DC-treated, OBP-702-treated, and combo-treated tumors. Data are expressed as the mean ± SD (*n* = 5). Statistical significance was determined using one-way ANOVA followed by Tukey’s comparison test. *P < 0.05; **P < 0.01; ***P < 0.001.

To evaluate the therapeutic potential of combination therapy with Ad-p53 DCs and OBP-702 (Supplementary Fig. 1C), we used CT26 murine CC cells with wild-type p53 protein due to weak p53 expression^18^. CT26 cells were infected with OBP-702 for 72 h, after which cell viability was analyzed using the XTT assay. OBP-702 infection significantly decreased the viability of CT26 cells in a dose-dependent manner (Fig. 2A). Next, CT26 cells were infected with OBP-702 for 24 and 72 h, and the expression levels of human p53 mRNA and protein were analyzed using real-time RT-PCR and Western blot analyses. OBP-702 infection induced the expression of human p53 mRNA and protein in p53-intact CT26 cells (Fig. 2B and C and Supplementary Fig. 5). These results suggest that CT26 cells are sensitive to OBP-702-mediated cytopathic activity and p53 activation.

To investigate the in vivo therapeutic potential of combination therapy with Ad-p53 DCs and OBP-702, subcutaneous murine CC tumor models with p53-intact CT26 cells were used. As 2 rounds of immunization with Ad-p53 DCs was shown to efficiently induce anti-p53 immune responses in mice^19^, Ad-p53 DCs were subcutaneously injected into the flanks of mice weekly for 4 weeks on days −21, −14, −7, and +6, before and after tumor inoculation (day 0) (Fig. 2D). CT26 tumor-bearing mice then received intratumoral injections of OBP-702 on days 7 and 10 after tumor inoculation (Fig. 2D). Combination therapy with Ad-p53 DCs and OBP-702 significantly suppressed tumor growth compared with the control and monotherapy groups (Fig. 2E and F). Tumor weight was significantly decreased in the combination therapy group compared with the control and monotherapy groups (Fig. 2G). These results suggest that combination treatment with OBP-702 enhances the antitumor efficacy of Ad-p53 DCs in p53-intact CT26 tumors.

### Enhancement of the antitumor immune response in CT26 tumors treated with Ad-p53 DCs and OBP-702

**Fig. 3:**
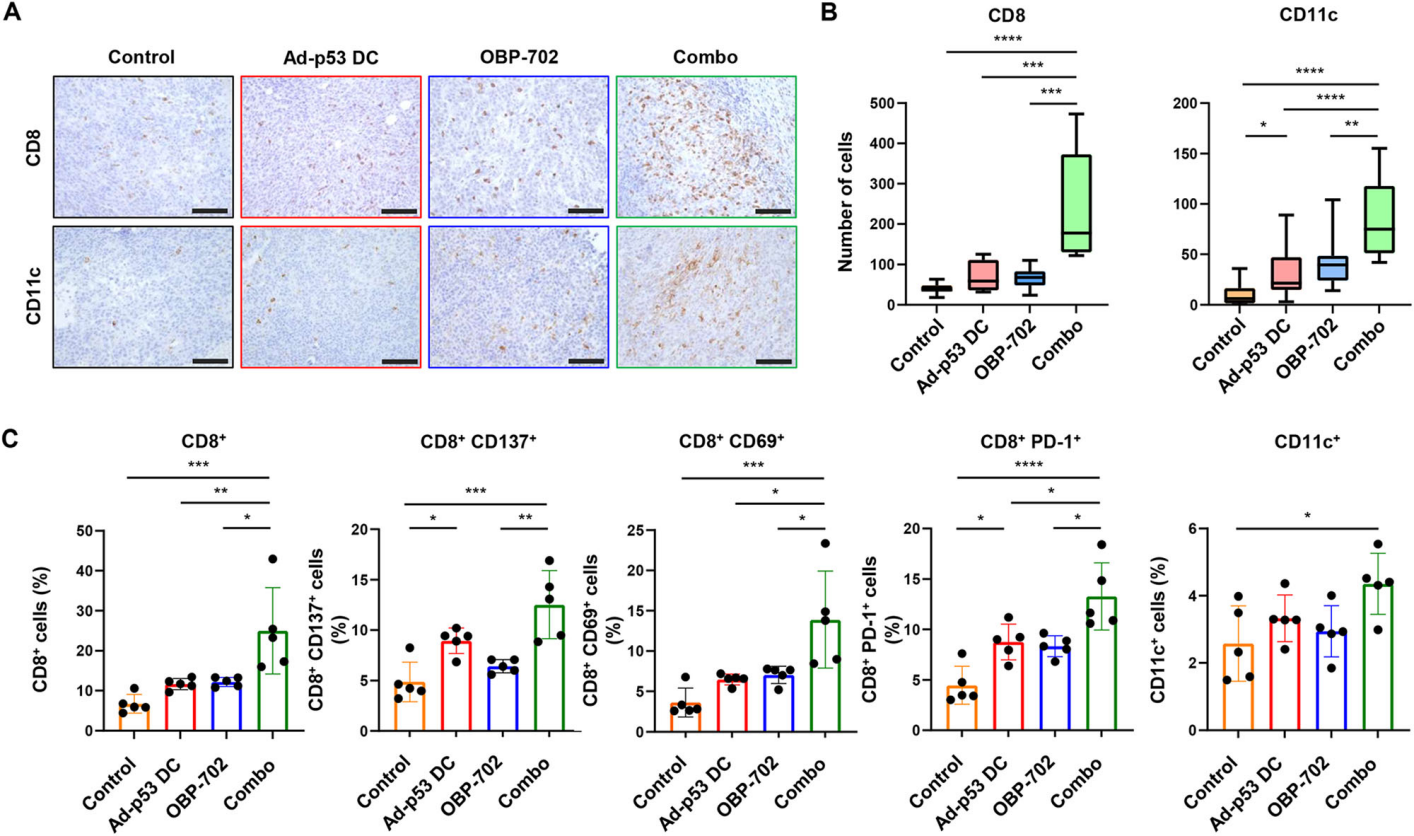
Immunohistochemical staining and flow cytometric analysis of CT26 tumors. **A** Representative photographs of immunohistochemical staining for CD8 + T cells and CD11c+ DCs in each group. Scale bars, 50 μm. **B** Percentage of CD8+ cells and CD11c+ cells calculated from five different randomly selected fields. **C** Percentage of CD8 + , CD8 + CD137 + , CD8 + CD69 + , CD8 + PD-1 + , and CD11c+ cells among live cells assessed using flow cytometry. Data are expressed as the mean ± SD (*n* = 5). Statistical significance was determined using one-way ANOVA followed by Tukey’s comparison test. *P < 0.05; **P < 0.01; ***P < 0.001; ****P < 0.0001.

To investigate whether combination therapy modulates immunity in the TME, the proportions of CD8 + T cells and CD11c+ DCs were analyzed using immunohistochemistry and flow cytometry (Supplementary Figs. 6 and 7). Immunohistochemistry analysis demonstrated that the numbers of CD8 + T cells and CD11c+ DCs were significantly higher in tumors treated with combination therapy compared with control or monotherapy-treated tumors (Fig. 3A and B). Flow cytometric analysis showed that combination therapy-treated tumors contained a significantly higher proportion of CD8+ CTLs and CD11c+ DCs compared with control and monotherapy-treated tumors (Fig. 3C).

We also examined the proportions of CD8 + CD137 + , CD8 + CD69 + , and CD8 + PD-1+ cells. CD137 is an antigen-specific T cell marker^20^, whereas CD69 is a memory T cell marker^21^, and PD-1 is an antigen-engagement T cell marker^22^. Combination therapy significantly increased the proportions of CD8 + CD137 + , CD8 + CD69 + , and CD8 + PD-1+ cells compared with the control and monotherapy-treated groups (Fig. 3C). These results suggest that combination therapy with OBP-702 enhances the antitumor efficacy of Ad-p53 DCs via tumor infiltration of CD8+ CTLs and CD11c+ DCs.

### OBP-702 enhances the antitumor efficacy of Ad-p53 DCs via activation of the antitumor immune response

**Fig. 4:**
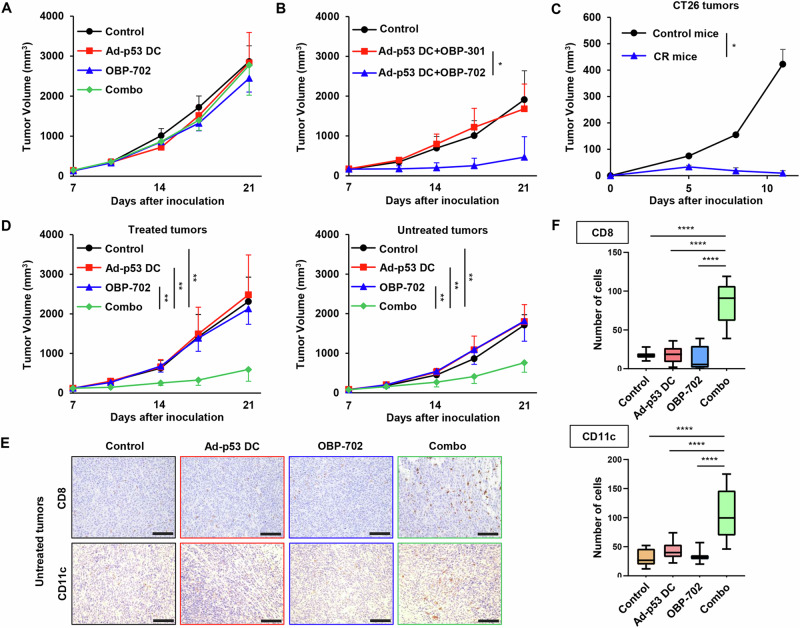
Role of the antitumor immune response in combination therapy. **A** Combined effect of Ad-p53 DC and OBP-702 in the CT26 tumor model using athymic nude mice. Data are expressed as the mean tumor volume ± SD (*n* = 5). **B** Combined effect of Ad-p53 DC and OBP-301 in the CT26 tumor model. Data are expressed as the mean tumor volume ± SD (*n* = 6). **C** Difference in the growth of CT26 tumors between control and complete response (CR) mice. Combination therapy-treated CR mice and control mice were subcutaneously inoculated with CT26 cells. Data are expressed as the mean ± SD (*n* = 3). **D** Growth curves for treated and untreated sites of control, Ad-p53 DC-treated, OBP-702-treated, and combination therapy (combo)-treated tumors in bilateral CT26 tumor models. Data are expressed as the mean ± SD (*n* = 5). **E** Representative photographs of immunohistochemical staining for CD8 + T cells and CD11c+ DCs in each group. Scale bars, 50 μm. **F** Percentage of CD8+ cells and CD11c+ cells calculated from five different randomly selected fields. Data are expressed as the mean ± SD (*n* = 5). Statistical significance was determined using one-way ANOVA followed by Tukey’s comparison test. *P < 0.05; **P < 0.01; ****P < 0.0001.

To evaluate the role of T cell-mediated antitumor immunity in combination therapy, we analyzed the effect of combination therapy using athymic nude mice lacking T cells. The antitumor efficacy of Ad-p53 DCs in combination with OBP-702 was diminished in athymic nude mice (Fig. 4A), suggesting that T cells are necessary for the combined effect of Ad-p53 DCs and OBP-702.

To investigate the role of p53-targeting CTLs in combination therapy, we analyzed the therapeutic potential of non-armed OBP-301 (Supplementary Fig. 1D) to enhance the antitumor efficacy of the Ad-p53 DC vaccine. However, non-armed OBP-301 did not enhance the antitumor effect of the Ad-p53 DC vaccine (Fig. 4B). These results suggest that OBP-702-mediated p53 activation in tumor cells plays an important role in promoting the antitumor effect of the Ad-p53 DC vaccine to induce p53-targeting CTLs.

To evaluate whether combination therapy induces systemic antitumor immunity, we conducted a rechallenge test using 3 mice that achieved CR after treatment with combination therapy and compared them with control mice. CT26 and 4T1 murine breast cancer cells were inoculated into the right and left flanks of mice, respectively. Although 4T1 cells developed comparable tumors between CR and control mice (Supplementary Fig. 8), CT26 cells developed tumors only in the control mice (Fig. 4C). These results suggest that combination therapy induces long-lasting antitumor immunity in mice showing a CR.

To further investigate whether combination therapy induces systemic antitumor immunity, we analyzed the abscopal effect of combination therapy using bilateral CT26 tumor models (Supplementary Fig. 9). CT26 cells were inoculated into the bilateral flanks of mice, and one side tumor was mock treated or treated with mono- or combination therapy (Supplementary Fig. 9). Combination therapy significantly suppressed the growth of both treated and untreated tumors (Fig. 4D). By contrast, monotherapy with Ad-p53 DCs or OBP-702 suppressed the growth of treated tumors but not untreated tumors (Fig. 4D). Immunohistochemistry demonstrated that combination therapy significantly increased the proportion of CD8 + T cells and CD11c+ DCs in untreated tumors (Fig. 4E and F). These results suggest that combination therapy exerts an abscopal effect in OBP-702-untreated CT26 tumors via induction of the antitumor immune response.

### Identification of p53-derived peptides commonly bound to MHC molecules on the surfaces of Ad-p53 DCs and OBP-702-infected tumor cells

**Fig. 5:**
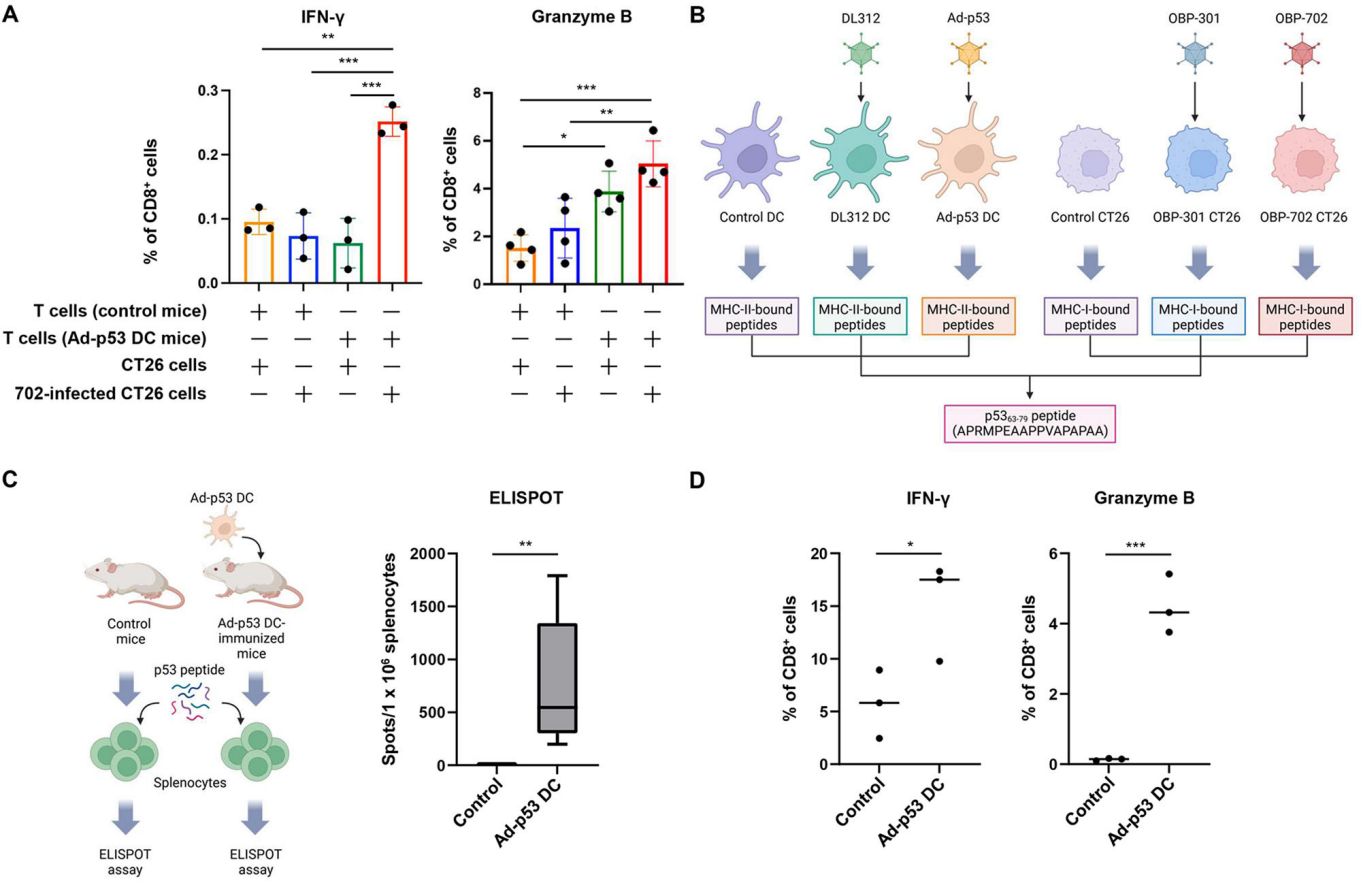
Immunopeptidomic identification of MHC-bound p53-derived peptides. **A** Splenocytes isolated from control and Ad-p53 DC-immunized mice were stimulated with IL-2 and co-cultured with OBP-702-infected and non-infected CT26 cells for 6 h and then subjected to FACS analysis. The percentage of IFN-γ+ and granzyme B+ cells among CD8+ cells was assessed by flow cytometry. Data are expressed as the mean ± SD (*n* = 3 or 4). **B** MHC-II-bound peptides were isolated from control, DL312-infected, and Ad-p53-infected DCs. MHC-I-bound peptides were isolated from control, OBP-301-infected, and OBP-702-infected CT26 cells. Human p53-derived p53_63-79_ peptide (APRMPEAAPPVAPAPAA) was commonly expressed in the context of MHC-I and MHC-II molecules. Figures were generated using BioRender. **C** Splenocytes isolated from control and Ad-p53 DC-immunized mice were incubated with p53 peptide for 72 h and subjected to ELISPOT assay. The number of spots per 1 × 10^6^ splenocytes is shown. Figures were generated using BioRender. Data are expressed as the mean ± SD (*n* = 3). **D** Splenocytes isolated from control and Ad-p53 DC-immunized mice were incubated with p53 peptide and incubated for 72 h. The percentage of IFN-γ+ and granzyme B+ cells among CD8+ cells was assessed by flow cytometry. Data are expressed as the mean ± SD (*n* = 3). The statistical significance of differences between two groups or four groups was determined using the Student *t*-test or one-way ANOVA followed by Tukey’s comparison test, respectively. *P < 0.05; **P < 0.01; ***P < 0.001.

As OBP-702 induces p53 overexpression in human and murine tumor cells^13,15^, OBP-702-infected tumor cells that overexpress p53 may be more sensitive to p53-targeting CTLs induced by Ad-p53 DCs than non-treated tumor cells. To evaluate whether Ad-p53 DC vaccine therapy induces CTLs targeting OBP-702-infected tumor cells, we isolated splenocytes from Ad-p53 DC-treated and untreated mice. Splenocytes were stimulated with IL-2 to induce CD8 + T cells. The resulting CD8 + T cells were co-cultured with OBP-702-infected and non-infected CT26 cells for 6 h, after which the proportions of CD8 + T cells expressing IFN-γ or granzyme B were determined by FACS analysis (Supplementary Fig. 10). CD8 + T cells obtained from Ad-p53 DC-immunized mice exhibited a significantly higher proportion of IFN-γ+ and granzyme B + CD8 + T cells than other groups when co-cultured with OBP-702-infected CT26 cells, but not when co-cultured with non-infected CT26 cells (Fig. 5A). These results suggest that Ad-p53 DC vaccine therapy induces CTLs targeting OBP-702-infected CT26 cells.

Ad-p53 DCs have been shown to present p53 peptides bound to MHC-I and MHC-II, resulting in the induction of p53-specific CD8 + T cells and CD4 + T cells, respectively^19^. To determine whether Ad-p53 DCs and OBP-702-infected tumor cells present p53 peptides bound to MHC molecules, we employed an immunopeptidomics approach using mass spectrometry^23^. MHC-I-bound peptides were obtained from control CT26 cells and CT26 cells infected with non-armed OBP-301 or OBP-702. MHC-II-bound peptides were isolated from control DCs and DCs transduced with control DL312 or Ad-p53^24^. All peptide sequences contained in human p53 protein were isolated using mass spectrometry. We then excluded p53 peptides commonly found in mouse p53 because we could not distinguish whether p53 peptides were derived from exogeneous human p53 or endogenous mouse p53 protein. Moreover, we excluded peptides bound to MHC-I molecules of control CT26 cells and MHC-II of control DCs because these peptides were derived from endogenous mouse p53 and not exogenous human p53 protein. The immunopeptidomics analysis demonstrated that OBP-702-infected CT26 cells and Ad-p53 DCs commonly exhibited MHC-bound p53_63-79_ peptide (APRMPEAAPPVAPAPAA) derived from human p53 protein (Fig. 5B and Supplementary Table 1). These results suggest that the human p53_63-79_ peptide commonly binds to MHC-I of OBP-702-infected tumor cells and MHC-II of Ad-p53 DCs.

To determine whether p53 peptides function as tumor antigens, we employed an ELISPOT assay using a mixture of synthetic p53 peptides: p53_63-70_ (APRMPEAA), p53_65-73_ (RMPEAAPPV), and p53_72-79_ (PVAPAPAA). Splenocytes isolated from Ad-p53 DC-immunized and control mice were stimulated with IL-2 to induce T cells and then co-cultured with the three different p53 peptides. Ad-p53 DC-immunized mice exhibited a CD8 + T cell response to the mixture of p53 peptides (Fig. 5C). These results suggest that OBP-702 induces the binding of p53 peptides to MHC-I molecules on the surface of virus-infected tumor cells, resulting in elimination of the tumor cells by Ad-p53 DC-mediated p53-related CTLs.

To determine whether Ad-p53 DC vaccine therapy induces CTLs targeting p53 peptides, we isolated splenocytes from Ad-p53 DC-treated and non-treated mice. The splenocytes were then stimulated with IL-2 to induce CD8 + T cells, which were co-cultured with the mixture of p53 peptides for 6 h, after which the proportions of CD8 + T cells expressing IFN-γ or granzyme B were determined by FACS analysis (Fig. 5D). CD8 + T cells obtained from Ad-p53 DC-immunized mice exhibited a significantly higher proportion of IFN-γ+ and granzyme B + CD8 + T cells than control cells when co-cultured with the mixture of p53 peptides (Fig. 5D). These results suggest that Ad-p53 DC vaccine therapy induces CTLs targeting p53 peptides.

### In vivo antitumor effect of Ad-p53 DC and OBP-702 combination therapy against p53-mutant MC38 tumors

**Fig. 6:**
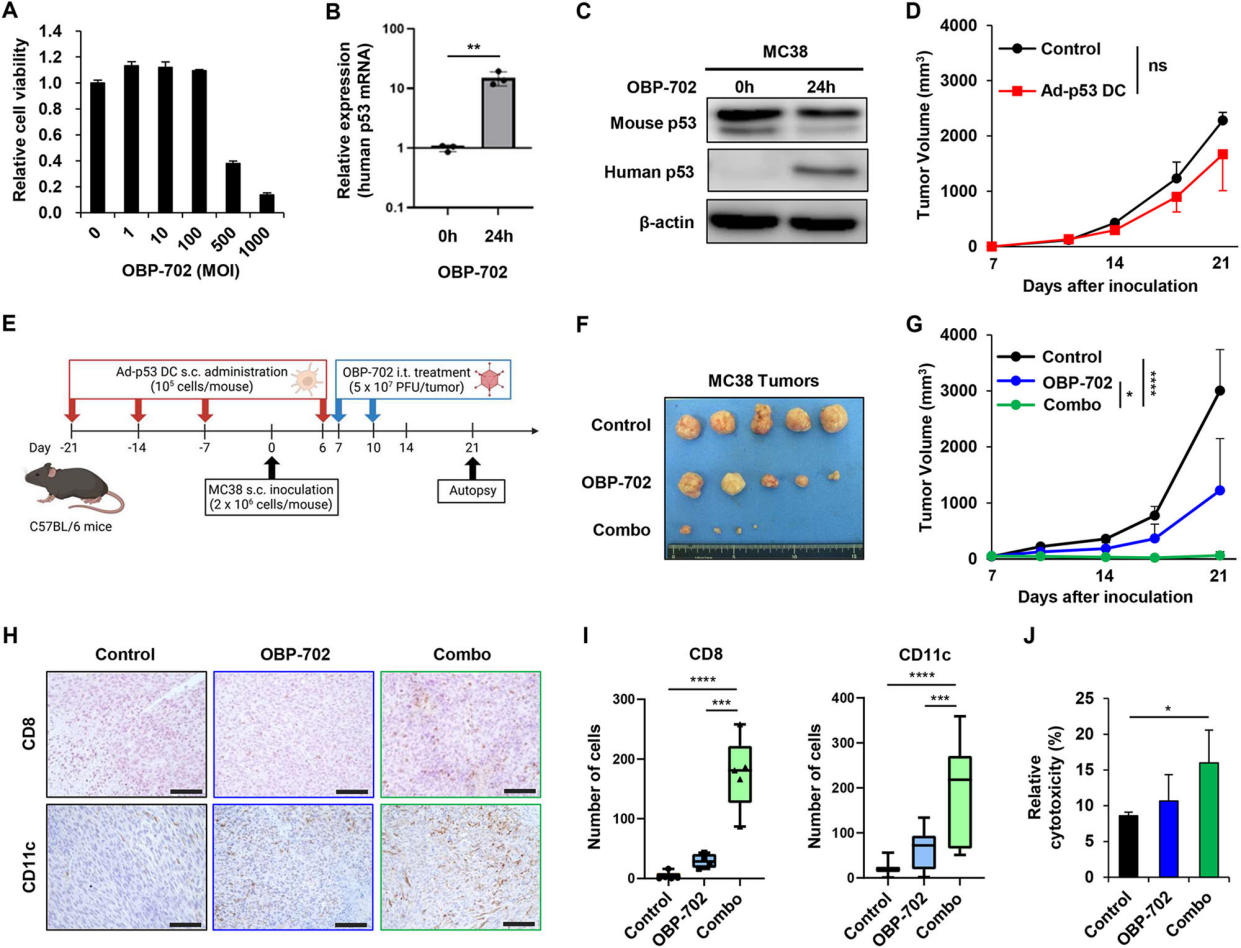
Combined effect of Ad-p53 DCs and OBP-702 in a p53-mutant murine MC38 tumor model. **A** Viability of MC38 cells was assessed using an XTT assay 3 days after OBP-702 treatment at the indicated MOIs. Cell viability was calculated relative to that of mock-infected cells, which were set as 1.0. **B** Expression of human p53 mRNA in MC38 cells before and after infection with OBP-702 (100 MOI) for 24 h. Data are expressed as mean ± SD (*n* = 3). **C** Whole-cell lysates of MC38 cells before and after infection with OBP-702 (100 MOI) for 24 h were subjected to Western blot analysis of mouse p53, human p53, and β-actin expression. **D** Growth curves of control and Ad-p53 DC-treated tumors. Data are expressed as mean ± SD (*n* = 3). **E** Schematic illustration of the MC38 tumor model experimental protocol. Figures were generated using BioRender. **F** Photographs of tumors in each group. **G** Growth curves of control, Ad-p53 DC-treated, OBP-702-treated, and combination therapy (combo)-treated tumors. **H** Representative photographs of immunohistochemical staining for CD8 + T cells and CD11c+ DCs in each group. Scale bars, 50 μm. **I** Percentage of CD8+ cells and CD11c+ cells calculated from five different randomly selected fields. Data are expressed as the mean ± SD (*n* = 5). **J** CD8 + T cells isolated from splenocytes of control and treated mice were co-cultured with MC38 cells for 24 h, and the amount of extracellular LDH released from tumor cells was determined. The statistical significance of differences between two groups or three groups was determined using the Student *t*-test or one-way ANOVA followed by Tukey’s comparison test, respectively. *P < 0.05; **P < 0.01; ***P < 0.001; ****P < 0.0001; ns, not significant.

To evaluate the therapeutic potential of combination therapy with Ad-p53 DCs and OBP-702 against another type of CC cells, we used MC38 mouse CC cells expressing mutant-type p53 protein^18^. MC38 cells were infected with OBP-702 for 72 h, after which cell viability was analyzed by XTT assay. OBP-702 infection significantly decreased the viability of MC38 cells in a dose-dependent manner (Fig. 6A). Next, MC38 cells were infected with OBP-702 for 24 h, and the expression levels of human p53 mRNA and protein were analyzed by real-time RT-PCR and Western blotting, respectively. OBP-702 infection induced the expression of human p53 mRNA and protein in p53-mutant MC38 cells (Fig. 6B and C**and** Supplementary Fig. 5). These results suggest that MC38 cells are sensitive to OBP-702-mediated cytopathic activity and p53 activation.

As MC38 cells are mutant p53-overexpressing CC cells, a p53-targeting DC vaccine alone may exhibit therapeutic potential for suppressing the growth of p53-mutant MC38 tumors by inducing p53-targeting CTLs^25^. The therapeutic potential of Ad-p53 DCs against p53-mutant CC tumors was investigated using subcutaneous murine CC tumor models with p53-mutant MC38 cells. Ad-p53 DCs were subcutaneously injected into the flanks of mice weekly for 4 weeks on days −21, −14, −7, and +6, before and after tumor inoculation (day 0). However, Ad-p53 DC monotherapy did not inhibit the growth of p53-mutant MC38 tumor cells (Fig. 6D). These results suggest that monotherapy with Ad-p53 DCs is not sufficient to suppress the growth of p53-mutant MC38 tumors.

To evaluate the in vivo therapeutic potential of combination therapy with Ad-p53 DCs and OBP-702 against p53-mutant CC tumors, we used subcutaneous murine CC tumor models with p53-mutant MC38 cells (Fig. 6E). Ad-p53 DCs were subcutaneously injected into the flanks of mice weekly for 4 weeks on days −21, −14, −7, and +6, before and after tumor inoculation (day 0). The MC38 tumor-bearing mice then received intratumoral injection of OBP-702 on days 7 and 10 after tumor inoculation. Combination therapy with Ad-p53 DCs and OBP-702 significantly suppressed tumor growth compared with the control and monotherapy groups (Fig. 6F and G). Immunohistochemistry analysis demonstrated that the numbers of CD8 + T cells and CD11c+ DCs were significantly higher in tumors treated with combination therapy compared with control or monotherapy-treated tumors (Fig. 6H and I). These results suggest that combination treatment with OBP-702 enhances the antitumor efficacy of Ad-p53 DCs against p53-mutant MC38 tumors.

To determine whether OBP-702-infected MC38 cells express p53 peptides on the surface of MHC molecules, MHC-I-bound peptides were isolated from OBP-702-infected MC38 cells. Immunopeptidomics analysis demonstrated that OBP-702-infected MC38 cells exhibited the p53_63-75_ peptide (APRMPEAAPPVAP) derived from human p53 protein, similar to OBP-702-infected CT26 cells (Supplementary Table 1). By contrast, control MC38 or OBP-301-infected MC38 cells did not express the p53_63-75_ peptide (APRMPEAAPPVAP). These results suggest that the p53_63-75_ peptide (APRMPEAAPPVAP) commonly binds to MHC-I molecules of OBP-702-infected MC38 and CT26 cells.

To determine whether combination therapy induces systemic antitumor immunity, we employed an LDH assay using splenocytes. Splenocytes isolated from control and combination therapy-treated mice were co-cultured with irradiated MC38 cells in the presence of IL-2 for 7 days. CD8 + T cells were then co-cultured with MC38 cells for 24 h, after which the amount of extracellular LDH released from the tumor cells was measured. The amount of LDH released was significantly higher in MC38 cells co-cultured with CD8 + T cells from combination therapy-treated mice compared with CD8 + T cells from control mice (Fig. 6J). These results suggest that combination therapy with OBP-702 enhances the therapeutic potential of Ad-p53 DCs in p53-mutant MC38 tumors via activation of the antitumor immune response.

## Discussion

DC-based vaccine therapy has been shown to promote the activation of tumor-targeting CTLs^1^. However, cold tumors are relatively resistant to DC vaccine therapy due to weak tumor immunogenicity, poor infiltration of CTLs, and formation of an immunosuppressive TME^26^. Therefore, combination immunotherapies that enhance tumor immunogenicity and the antitumor immune response are needed to increase the therapeutic potential of DC vaccine therapy. In this study, we demonstrated that combination therapy with Ad-p53 DCs and OBP-702 markedly reduced the growth of murine CC tumor models with p53 wild-type and p53 mutant tumor cells by activating tumor-infiltrating CD8+ CTLs and CD11c+ DCs. Combination therapy with OBP-702 also promoted an abscopal effect in OBP-702-untreated tumors via activation of the antitumor immune response. Immunopeptidomics analyses demonstrated that OBP-702-infected tumor cells express MHC-I-bound p53_63-79_ peptide (APRMPEAAPPVAPAPAA) derived from human p53 protein commonly expressed on MHC-II molecules of Ad-p53 DCs. Ad-p53 DC-immunized mice exhibited the presence of p53_63-79_ peptide-targeting CTLs, which preferentially eliminated OBP-702-infected tumor cells. Thus, combination therapy with Ad-p53 DCs and OBP-702 may be a promising antitumor strategy for inducing a strong antitumor effect by activating the presentation of p53 peptides in tumor cells and recruiting p53 peptide-targeting CTLs to tumors.

Vaccine therapy using Ad-p53 DCs is a promising antitumor strategy for eliminating p53-expressing cancer cells via the induction of p53-targeting CTLs^5^. However, the antitumor efficacy of p53-targeting CTLs induced by Ad-p53 DC vaccine therapy is limited due to weak p53 expression by p53-intact tumor cells. In this study, combination therapy with OBP-702 significantly enhanced the therapeutic potential of Ad-p53 DC vaccine therapy in a p53-wild-type CT26 tumor model. These findings suggest that OBP-702-mediated p53 activation in p53-intact tumor cells is a promising approach for enhancing the antitumor efficacy of Ad-p53 DC vaccine therapy by sensitizing tumor cells to p53-targeting CTLs. In terms of the approach for promoting p53 activation in p53-intact tumor cells, MDM2 inhibitors have been shown to activate p53 expression in tumor cells, thereby contributing to the activation of antitumor immunity^27,28^. Chemotherapy has been shown to activate p53 expression in tumor cells, which promotes sensitization to Ad-p53 DC vaccine therapy^29^. However, systemic MDM2 inhibitor administration along with chemotherapy may activate p53 expression not only in tumor cells but also normal cells, resulting in an increased risk of adverse events. Therefore, OBP-702-mediated tumor-specific p53 activation may be a more useful strategy than conventional therapies to increase sensitivity to p53-targeting CTLs induced by Ad-p53 DC vaccine therapy.

Ad-p53 DC vaccine therapy has been shown to induce wild-type p53 epitope-targeting CTLs^5^, which eliminate p53-expressing tumor cells. In this study, combination treatment with p53-armed OBP-702, but not non-armed OBP-301, significantly enhanced the therapeutic potential of Ad-p53 DC vaccine therapy in a p53-intact CT26 model. Moreover, OBP-702-mediated expression of human p53 could sensitize p53-mutant MC38 tumors to Ad-p53 DC vaccine therapy. These findings suggest that OBP-702 induces the presentation of tumor cell human p53 epitopes to increase tumor cell sensitivity to human p53-targeting CTLs. OBP-702-infected CT26 and MC38 cells commonly expressed the MHC-I-bound p53_63-75_ epitope (APRMPEAAPPVAP), which was also expressed on MHC-II molecules of Ad-p53 DCs. As previous report have shown that p53_65-73_ peptide (RMPEAAPPV) is an HLA-A2-restricted wild-type p53 epitope on the surface of human cancer cells^30^, the p53_65-73_ epitope may be a potent therapeutic target for Ad-p53 DC-induced p53-targeting CTLs in both mice and humans. We previously demonstrated that Ad-p53 DCs induce p53-targeting CTLs to target human p53-overexpressing tumor cells^31^. Thus, further experiments are needed to evaluate the role of the p53_65-73_ peptide in Ad-p53 DC and OBP-702 combination therapy against human CC tumors.

Combination therapy with Ad-p53 DCs and OBP-702 induced an abscopal effect in untreated CT26 tumors via induction of tumor-infiltrating CD8+ CTLs and CD11c+ DCs (Fig. 4D-F). Moreover, combination therapy-treated CR mice were resistant to re-inoculation with CT26 cells. These findings suggest systemic antitumor immunity is activated in combination therapy-treated mice. Examining the abscopal effect in bilateral CT26 tumor models, Ghaffari-Nazari et al. demonstrated that radiation induces the abscopal effect via induction of tumor-infiltrating CD8+ CTLs^32^. Yu et al. showed the therapeutic potential of microwave ablation in inducing the abscopal effect via induction of CD8 + T cells and CD103+ DCs^33^. We demonstrated that intratumoral injection of a fiber-modified OBP-301 variant (OBP-502) induces the abscopal effect via the promotion of immunogenic cell death and the release of CCL5 and CXCL10^12^. Recently, we reported that intratumoral injection of OBP-702 induces long-lasting antitumor immunity involving CD8+ resident memory T cells in murine pancreatic tumor models^34^. Therefore, combination therapy with Ad-p53 DCs and OBP-702 may induce systemic antitumor immunity by promoting tumor-infiltration by DCs, CTLs, and resident memory T cells.

Ad-p53 DCs were subcutaneously administered to promote the activation of p53-targeting CTLs as vaccine therapy. Regarding the routes of DC administration, Zhou et al. demonstrated that intratumoral administration of CD103+ DCs is a promising approach to induce systemic and long-lasting tumor-specific CTLs^35^. We also previously showed that intratumoral administration of Ad-p53 DCs exhibits the antitumor effect in murine melanoma tumor models via induction of tumor-specific CTLs^36^. These findings suggest that intratumoral administration of Ad-p53 DCs is a promising strategy to induce tumor-specific CTLs. However, in this study, we evaluated the therapeutic potential of intratumoral administration of OBP-702 in combination with Ad-p53 DC vaccine therapy to induce p53-targeting CTLs. To avoid the direct antitumor effect of Ad-p53 DCs in combination therapy, we selected the subcutaneous administration of Ad-p53 DCs to induce p53-targeting CTLs in mice. Intratumoral administration of Ad-p53 DCs may be superior to subcutaneous injection of Ad-p53 DCs to induce profound antitumor immunity in combination therapy with OBP-702.

Murine CC tumor models using CT26 and MC38 cells were used to evaluate the combined effect of Ad-p53 DCs and OBP-702 (Figs. 2 and 6). We recently demonstrated that OBP-702 exhibits the antitumor effect against murine pancreatic ductal adenocarcinoma (PDAC) tumors via induction of the antitumor immune response^15^. These findings suggest that combination therapy with Ad-p53 DCs and OBP-702 induces profound antitumor effect against murine PDAC tumors. However, as the expression levels of MHC-I molecules are reduced in PDAC cells more frequently than CC cells^37^, MHC-I downregulation may hamper the antitumor effect of combination therapy with Ad-p53 DCs and OBP-702 in PDAC tumors. Therefore, further experiments are needed to evaluate the combined effect of Ad-p53 DCs and OBP-702 in murine PDAC tumor models.

In conclusion, we demonstrated that the p53-armed, oncolytic adenovirus OBP-702 enhances the antitumor efficacy of Ad-p53 DC vaccine therapy in murine CC tumor models by inducing the presentation of p53 peptides bound to MHC molecules on the surface of tumor cells, thereby sensitizing tumor cells to p53-targeting CTLs. Combination therapy involving Ad-p53 DCs and OBP-702 induced an abscopal effect against OBP-702-untreated tumors by activating the antitumor immune response. Taken together, our data indicate that combination therapy with p53-armed OBP-702 provides a novel antitumor platform for enhancing the efficacy of Ad-p53 DC vaccine therapy, in turn activating a strong antitumor immune response.

## Methods

### Cell lines

CT26 murine CC cells and 4T1 breast cancer cells derived from BALB/c mice were obtained from the American Type Culture Collection (Manassas, VA, USA). MC38 murine CC cells derived from C57BL/6 mice were obtained from Kerafast (Boston, MA, USA). CT26 and MC38 cells express wild-type and mutant p53 protein, respectively^18^. CT26 cells were maintained in RPMI-1640 medium supplemented with 10% fetal bovine serum (FBS). MC38 cells were maintained in Dulbecco’s modified Eagle’s medium supplemented with 10% FBS. All media were supplemented with 100 U/mL penicillin and 100 μg/mL streptomycin.

### Recombinant adenoviruses

The recombinant, telomerase-specific, replication-competent adenovirus OBP-301 (suratadenoturev), in which the promoter element of the *human telomere reverse transcriptase* (*hTERT*) gene drives expression of the *E1A* and *E1B* genes, was previously constructed and characterized^8,9^ (Supplementary Fig. 1D). For tumor-specific induction of exogeneous p53 expression by OBP-301, we generated OBP-702 by inserting a human wild-type *p53* gene expression cassette derived from the Egr-1 promoter into the E3 region of OBP-301^13^ (Supplementary Fig. 1C). The replication-deficient, human wild-type p53-expressing adenovirus Ad-p53 was used to transduce DCs to express p53 protein (Supplementary Fig. 1A). The replication-deficient *E1A*-deleted adenovirus DL312 (Supplementary Fig. 1B) was used to obtain DL312 DCs as a control for Ad-p53 DCs. Recombinant viruses were purified by ultracentrifugation using cesium chloride step gradients, and virus titers were determined by plaque-forming assay using 293 cells. Adenoviruses were stored at −80°C.

### Preparation of Ad-p53 DCs

Bone marrow-derived cells (BMDCs) were isolated from the femurs of BALB/c and C57BL/6 J mice (CLEA Japan, Tokyo, Japan). Mice were euthanized by cervical dislocation after general anesthesia with 2% isoflurane. BMDCs were incubated with recombinant mouse granulocyte-macrophage colony-stimulating factor (GM-CSF) (G0282; Sigma-Aldrich, St. Louis, MO, USA) at a concentration of 40 ng/mL and recombinant mouse interleukin (IL)-4 (SRP3211; Sigma-Aldrich) at a concentration of 10 ng/mL for 5 days. The BMDCs were then sham-treated or further infected with Ad-p53 or DL312 at a multiplicity of infection (MOI) of 100 plaque-forming units (PFUs)/cell for 2 days to obtain Ad-p53 DCs or DL312 DCs.

### Quantitative real-time PCR

Total RNA was extracted from control DCs, DL312 DCs, and Ad-p53 DCs using a miRNeasy Mini kit (Qiagen, Valencia, CA, USA). cDNA was synthesized from 100 ng of total RNA using a TaqMan reverse transcription kit (Applied Biosystems, Foster City, CA, USA). Human p53 and glyceraldehyde-3-phosphate dehydrogenase (GAPDH) mRNA expression was assessed using a StepOne-Plus^TM^ real-time PCR system (Applied Biosystems). The following primers were used: TP53 (Hs01034253_m1), Gapdh (Mm99999915_g1), and Actb (Mm02619580_g1). The 2^−ΔΔCt^ method was used to calculate relative expression levels of human p53 mRNA after normalization with reference to the expression of GAPDH mRNA (Fig. 1B) or Actb mRNA (Supplementary Fig. 2).

### Immunocytochemistry

Ad-p53 DCs seeded in a 12-well plate at a density of 1 × 10^6^ cells/well were fixed with 4% paraformaldehyde and permeabilized with methanol. Cells were placed on slides and stained overnight with the primary antibody against human p53 (1:800, 2527; Cell Signaling Technology, Beverly, MA, USA). After washing with phosphate-buffered saline (PBS), the slides were incubated with Alexa Fluor 647-labeled secondary antibody (1:500, A-21245; Invitrogen, Carlsbad, CA, USA). DAPI was used to identify nuclei. Slides were covered and photographed using a confocal laser scanning microscope (IX83; Olympus, Tokyo, Japan).

### Flow cytometric analysis

Expression of the DC maturation markers CD11c, CD86, CD103, CCR7, and MHC-II was evaluated in control DCs, DL312 DCs, and Ad-p53 DCs using flow cytometry. The mean fluorescence intensity (MFI) of each protein was determined by calculating the difference between the MFI of antibody- and control IgG-treated cells. Tumor-infiltrating immune cells were evaluated by monitoring CD45+ hematopoietic cells, CD8 + T cells, and CD11c+ DCs using flow cytometry. Flow cytometry was also used to evaluate the phenotypes of CD8 + T cells by determining the proportions of CD8 + CD137+ cells, CD8 + CD69+ cells, and CD8+programmed cell death 1 (PD-1)+ cells. Single-cell suspensions were stained with fluorescence-labeled primary antibodies. Details regarding the antibodies used for flow cytometry are shown in Supplementary Table 2. Isotype control IgG was used as a negative control. Cells were assayed for fluorescence using BD FACSLyric or FACSAria flow cytometers (BD Biosciences, San Jose, CA, USA), and data were analyzed using FlowJo software, version 7.6.5.

### Preparation and biodistribution of Ad-GFP-DCs

Bone marrow-derived cells (BMDCs) isolated from the femurs of BALB/c mice were incubated with recombinant mouse GM-CSF (G0282; Sigma-Aldrich) at a concentration of 40 ng/mL and recombinant mouse IL-4 (SRP3211; Sigma-Aldrich) at a concentration of 10 ng/mL for 5 days and further infected with replication-deficient green fluorescent protein (GFP)-expressing adenovirus Ad-GFP at a MOI of 100 PFUs/cell for 2 days to obtain Ad-GFP-DCs. To evaluate the biodistribution of DCs after inoculation, Ad-GFP-DCs or control DCs were subcutaneously administered to the left footpad of mice. After 24 h, the left inguinal lymph nodes were dissected, and frozen sections were prepared and analyzed to detect GFP signals using a fluorescence microscope (IX83; Olympus).

### Cell viability assay

Cells were seeded in 96-well plates at a density of 1 × 10^3^ cells/well 24 h before virus infection. The cells were then infected with OBP-301 or OBP-702 at a MOI of 0, 1, 5, 10, 50, or 100 PFUs/cell. Cell viability was determined 3 days after virus infection using a TACS® XTT Cell Proliferation Assay (R&D Systems, Minneapolis, MN, USA) according to the manufacturer’s protocol.

### Western blot analysis

Cells were seeded in a 100-mm dish at a density of 1 × 10^6^ cells/dish 24 h before virus infection. The cells were then infected with OBP-301 or OBP-702 at a MOI of 0, 10, or 100 PFUs/cell. On day 2 after virus infection, whole-cell lysates were prepared in lysis buffer (50 mM Tris-HCl [pH 7.4], 150 mM NaCl, 1% Triton X-100) supplemented with protease inhibitor cocktail (Complete Mini; Roche, Indianapolis, IN, USA). Proteins were electrophoresed on 10-15% sodium dodecyl sulfate-polyacrylamide gels and transferred onto polyvinylidene difluoride membranes (Hybond-P; GE Health Care, Buckinghamshire, UK). The membranes were blocked with Blocking-One (Nacalai Tesque, Kyoto, Japan) at room temperature for 30 min. The following primary antibodies were used: mouse anti-p53 monoclonal antibody (mAb) (1:1000, 18032; Cell Signaling Technology), rabbit anti-p53 mAb (rodent specific) (1:1000, 32532; Cell Signaling Technology), and mouse anti-β-actin mAb (1:5000, A5441; Sigma-Aldrich). Horseradish peroxidase-conjugated antibodies against rabbit IgG (1:5000, NA934; GE Healthcare) or mouse IgG (1:2500, NA931; GE Healthcare) were used as secondary antibodies. Immunoreactive bands on blots were visualized using enhanced chemiluminescence substrate (ECL Prime; GE Healthcare).

### In vivo subcutaneous CT26 and MC38 tumor models

Animal experimental protocols were approved by the Ethics Review Committee for Animal Experimentation of Okayama University School of Medicine (OKU-2021762). BALB/c mice, C57BL/6 J mice, and BALB/c-nu/nu athymic nude mice were purchased from CLEA Japan. All mice were 6-week-old females and maintained under specific pathogen-free conditions. Ad-p53 DCs were administered subcutaneously every 7 days for 3 times on days −21, −14, and −7 before tumor inoculation and one time on day 6 after tumor inoculation under general anesthesia with 2% isoflurane. On day 0, BALB/c and C57BL/6 J mice were inoculated in the right flank with CT26 cells (2 × 10^6^ cells/mouse) or MC38 cells (2 × 10^6^ cells/mouse), respectively, under general anesthesia with 2% isoflurane. Growth of CT26 tumor cells was compared among 4 groups: 1) control group (*n* = 5), 2) Ad-p53 DC monotherapy group (*n* = 5), 3) OBP-702 monotherapy group (*n* = 5), and 4) combination therapy group (*n* = 5). Growth of MC38 tumor cells was compared among 2 groups: 1) control group (*n* = 5) and 2) Ad-p53 DC monotherapy group (*n* = 5) or 3 groups: 1) control group (*n* = 5), 2) OBP-702 monotherapy group (*n* = 5), and 3) combination therapy group (*n* = 5). When tumors reached approximately 5-7 mm in a diameter, OBP-702 was administered intratumorally every 3 days for 2 times on days 7 and 10. The perpendicular diameter of each tumor was then measured twice per week, and tumor volume was calculated as follows: tumor volume (mm^3^) = *a* × *b*^2^ × 0.5, where *a* represents the longest diameter, *b* represents the shortest diameter, and 0.5 is a constant used to calculate the volume of an ellipsoid. Mice were euthanized by cervical dislocation after general anesthesia with 2% isoflurane.

To investigate the role of T cells in combination therapy, BALB/c-nu/nu athymic nude mice were treated with Ad-p53 DCs or/and OBP-702 according to the same experimental protocol (*n* = 5 in each group). To evaluate the role of non-armed OBP-301 in combination therapy, BALB/c mice bearing CT26 tumors were treated with Ad-p53 DCs and OBP-301 or OBP-702 according to the same experimental protocol (*n* = 5 in each group). To further evaluate the potential of combination therapy to induce systemic antitumor immunity, we conducted a rechallenge experiment using complete response (CR) mice treated with combination therapy and non-treated mice (*n* = 3 in each group). After a 3-week interval for tumor eradication, CT26 cells (2 × 10^6^ cells/site) or 4T1 breast cancer cells (2 × 10^6^ cells/site) were re-inoculated in the right or left flank of CR and control mice under general anesthesia with 2% isoflurane. Tumor size was monitored twice each week until 14 days after tumor inoculation. To further investigate the abscopal effect of combination therapy, CT26 cells (2 × 10^6^ cells/site) were inoculated into the bilateral flanks of 6-week-old female BALB/c mice under general anesthesia with 2% isoflurane. When tumors reached approximately 5-7 mm in diameter, the tumors were injected with a 20-μL volume of solution containing OBP-702 (*n* = 5) at a dose of 1 × 10^8^ PFUs or with PBS (*n* = 5) once each week for three cycles. Tumor size was monitored twice each week until 21 days after tumor inoculation. Mice were euthanized by cervical dislocation after general anesthesia with 2% isoflurane.

### Immunohistochemistry

Tumor tissue sections (2 μm) were deparaffinized with xylene and rehydrated using a graded ethanol series. Endogenous peroxidases were blocked by incubation with 3% H_2_O_2_ for 10 min, and the sections were subjected to antigen retrieval by microwaving in citrate buffer or EDTA buffer for 14 min. Sections were incubated with primary antibody for 1 h and then with peroxidase-linked secondary antibody for 30 min at room temperature. Rabbit anti-CD8a mAb (1:100, 4SM15; eBioscience, San Diego, CA, USA) and rabbit anti-CD11c mAb (1:350, 97585; Cell Signaling Technology) were used as primary antibodies. The enzyme substrate 3,3′-diaminobenzidine (Dako) was used for visualization, and sections were counterstained with Meyer’s hematoxylin. The number of positive cells was determined from four randomly selected nonoverlapping fields with an abundance of cells.

### Intracellular staining of interferon-γ and granzyme B

To evaluate the proportion of CD8 + T cells targeting OBP-702-infected tumor cells, we isolated splenocytes from control and Ad-p53 DC-immunized mice. The splenocytes were stimulated with mouse recombinant IL-2 (212-12; PeproTech, Inc., Cranbury, NJ, USA) at a concentration of 20 IU/mL for 7 days to induce CD8 + T cells. The IL-2-treated splenocytes were then co-cultured with OBP-702-infected or non-infected CT26 cells at a ratio of 1:5 for 6 h and analyzed using flow cytometry. Monensin solution (420701; BioLegend) was added to stop the secretion of intracellular proteins 1 h after co-culture. Mixtures of splenocytes and tumor cells were filtered through a 40-μm filter into a 50-mL conical tube and centrifuged at 400 *g* for 5 min at 4 °C. After washing with PBS, the cell pellets were resuspended in 2 mL of red blood cell lysis buffer (420302; BioLegend) and incubated on ice for 5 min; the reaction was stopped by the addition of 10 mL of PBS. The percentages of CD8 + T cells expressing interferon-γ or granzyme B were determined by flow cytometry.

### Immunopeptidomics

MHC-bound peptides were isolated using a Piece Classic IP kit (26146; Thermo Fisher Scientific, Waltham, MA, USA) according to the manufacturer’s instructions. MHC-I-bound peptides were immunoaffinity purified from CT26 cells and MC38 cells mock treated and treated with OBP-301 and OBP-702 using mouse anti-MHC-I mAb (10 μg/sample, BE0077; BioXCell, Lebanon, NH, USA). MHC-II-bound peptides were immunoaffinity purified from control DCs, DL312 DCs, and Ad-p53 DCs using rat anti-MHC-II mAb (10 μg/sample, 14-5321-82; Invitrogen). Eluted MHC-bound peptides were desalted using Pierce C18 Spin Columns (89870; Thermo Fisher Scientific) and Amicon Ultra (Merck Millipore, Billerica, MA, USA). For LC-MS analysis of MHC-I peptides, each dry sample was dissolved in 10 μL of 0.1% formic acid by dispensing/aspirating 20 times using a micropipette.

### Synthetic peptides

The p53_63-70_ (APRMPEAA), p53_65-73_ (RMPEAAPPV), and p53_72-79_ (PVAPAPAA) peptides were obtained from Cosmo Bio Co. Ltd. (Tokyo, Japan) and dissolved and diluted in 0.1% trifluoroacetic acid.

### Enzyme-linked immunosorbent spot (ELISPOT) assay

A BDTM ELISPOT set (BD Biosciences) was used as an interferon (IFN)-c ELISpot assay. Splenocytes (1 × 10^6^/well) were stimulated with each peptide (40 ng/µL) in 200 μL of RPMI-1640 medium supplemented with 10% FBS, penicillin, and streptomycin at 37 °C for 72 h. The spot count in each well with confluence represented the spot count for the non-confluent area in each well. The confluent area was omitted from the evaluation. The following equation was used to estimate the total spot number in each well with confluence: total spot number = spot count + 2 × (spot count × percent confluence/[100% − percent confluence]).

### LDH assay

Splenocytes isolated from control and treated mice were co-cultured with irradiated MC38 cells in the presence of IL-2 for 7 days. The splenocytes were then treated with red blood cell lysis buffer (BioLegend) and a debris removal solution kit (Miltenyi Biotec, Bergisch Gladbach, Germany). Next, the splenocytes were co-cultured with MC38 cells irradiated with 100 Gy and incubated with INF-γ (100 U/mL) (Wako Pure Chemical Industries, Osaka, Japan) for 5 days for stimulation. After CD8-positive selection using CD8 (TIL) MicroBeads (Miltenyi Biotec) and LS Columns (Miltenyi Biotec), MC38 cells (target) were incubated with CD8+ cells (effector) for 4 h. Finally, CD8 + T cells isolated by CD8 beads selection were co-cultured with MC38 cells for 24 h, and the amount of extracellular LDH released from tumor cells was determined using a Cytotoxicity Detection Kit Plus (4744934001; Roche).

### Statistical analyses

Statistical analyses were performed using JMP software, version 14.3.0, and GraphPad Prism (version 8.0) software was used to create graphics. Data are presented as mean ± SD. Mean differences were compared using the Student *t*-test for 2 groups and one-way ANOVA with Tukey’s post-test for multiple groups. Spearman’s correlation coefficients were used to assess correlations between parameters. *P* values < 0.05 were considered statistically significant.

## Supplementary information

Supplementary Information

## Data Availability

All data generated or analyzed during this study are included in the main text or supplemental information. Further enquiries are directed to the corresponding author.
