# Supplementary material for: Oncolytic virus-mediated p53 activation boosts the antitumor immunity of a p53-transduced dendritic cell vaccine
Source: NPJ Vaccines. 2025 Jul 19;10:158. doi: 10.1038/s41541-025-01219-5 (PMC12276290; doi:10.1038/s41541-025-01219-5)
Supplement: Supplementary file 1 — Supplementary Information [file 41541_2025_1219_MOESM1_ESM.pdf]

Supplementary Information

Oncolytic virus-mediated p53 activation boosts the antitumor immunity of a p53-transduced dendritic cell vaccine

Motohiko Yamada[1], Hiroshi Tazawa[1,2], Kanto Suemori[1], Naohiro Okada[1], Yoshinori Kajiwara[1], Ryohei Shoji[1], Yasuo Nagai[1], Hiroaki Inoue[1], Naoyuki Hashimoto[1], Nobuhiko Kanaya[1], Satoru Kikuchi[1], Shinji Kuroda[1], Hiroyuki Michiue[3], Yasuo Urata[4], Shunsuke Kagawa[1], Toshiyoshi Fujiwara[1]

[1] Department of Gastroenterological Surgery, Okayama University Graduate School of Medicine, Dentistry and Pharmaceutical Sciences, Okayama 700-8558, Japan;
[2] Center for Innovative Clinical Medicine and [3] Neutron Therapy Research Center, Okayama University Hospital, Okayama 700-8558, Japan.
[4] Oncolys BioPharma, Inc., Tokyo 105-0001, Japan.

Supplementary Figure 1
Schematic diagrams of the structures of the replication-deficient adenoviruses (Ad-p53, DL312) and telomerase-specific replication-competent oncolytic adenoviruses (OBP-702, OBP-301).

Supplementary Figure 2
Expression of human p53 mRNA in control DCs and DCs infected with DL312 or Ad-p53 for 48 h.

Supplementary Figure 3
Gating strategy for DCs in FACS analysis.

Supplementary Figure 4
Evaluation of GFP+ cells at inguinal lymph nodes of Ad-GFP DC-treated mice.

Supplementary Figure 5
Full images of Figs. 2C and 6C.

Supplementary Figure 6
Gating strategy for CD8+ T cells in FACS analysis.

Supplementary Figure 7
Gating strategy for CD11c+ DCs in FACS analysis.

Supplementary Figure 8
Comparable growth of 4T1 tumors between control and CR mice.

Supplementary Figure 9
Schematic illustration of the experimental protocol of bilateral CT26 tumor models.

Supplementary Figure 10
Gating strategy for IFN-γ+ and granzyme B+ T cells in FACS analysis.

Supplementary Table 1

Identification of p53 peptides that were commonly expressed on the MHC molecules of Ad-p53 DCs and OBP-702-infected tumor cells.

Supplementary Table 2

Primary antibodies used for flow cytometry.

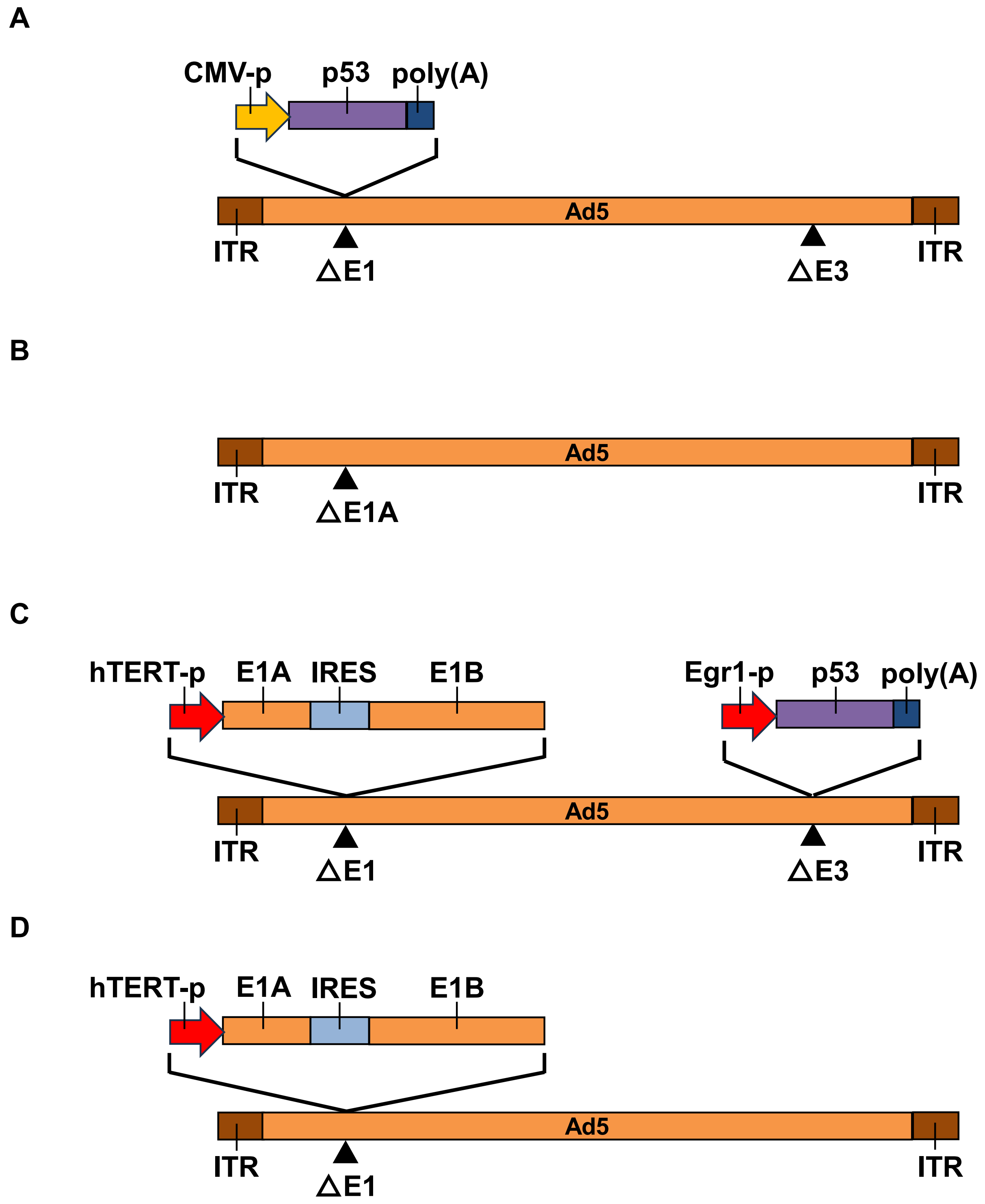

Supplementary Figure 1. Schematic diagrams of the structures of the replication-deficient adenoviruses (Ad-p53, DL312) and telomerase-specific replication-competent oncolytic adenoviruses (OBP-702, OBP-301). A, Ad-p53 is replication-deficient *E1/E3*-deleted adenovirus, in which the *CMV* promoter drives expression of the human wild-type p53 gene. **B**, DL312 is replication-deficient *E1A*-deleted adenovirus. **C**, OBP-702 is a p53-armed telomerase-specific, replication-competent oncolytic adenovirus, in which the *hTERT* promoter drives expression of the *E1A* and *E1B* genes and *Egr1* promoter drives expression of the human wild-type p53 gene. **D**, OBP-301 is a non-armed telomerase-specific, replication-competent oncolytic adenovirus, in which the *hTERT* promoter drives expression of the *E1A* and *E1B* genes. Ad5: adenovirus serotype 5; CMV: cytomegalovirus; hTERT: human telomere reverse transcriptase; IRES: internal ribosome entry site; ITR: inverted terminal repeat.

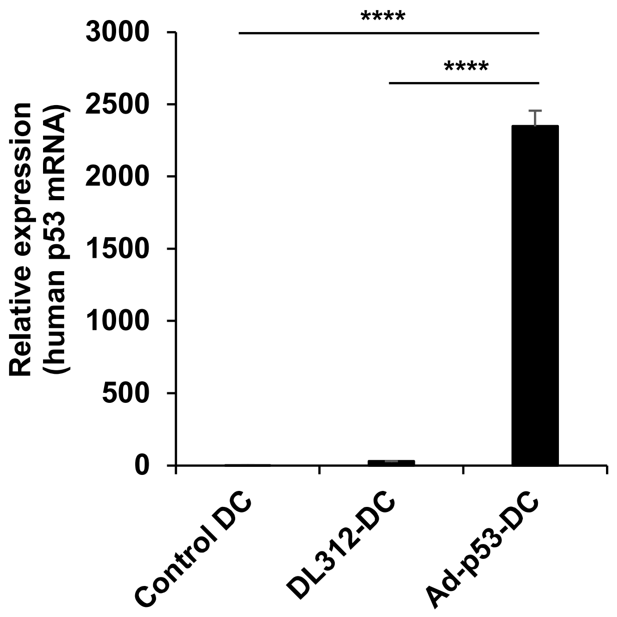

Supplementary Figure 2. Expression of human p53 mRNA in control DCs and DCs infected with DL312 or Ad-p53. β-actin was used as a control gene. The statistical significance of differences between three groups was determined using one-way ANOVA followed by Tukey's comparison test. ****, *P*<0.0001.

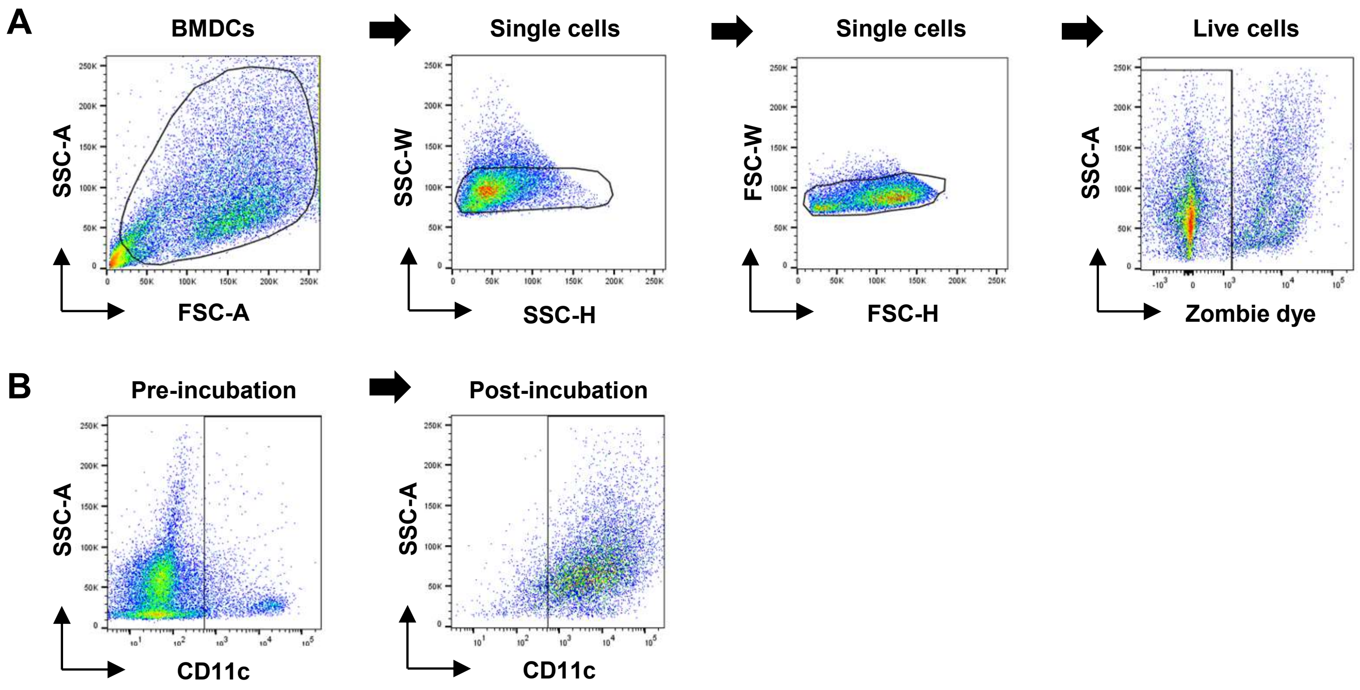

Supplementary Figure 3. Gating strategy for DCs in FACS analysis. A, Representative FSC, SSC, and zombie dye plots with gating for bone marrow-derived cells (BMDCs), single cells, and live cells. **B**, Representative CD11c plots with gating for DCs pre- and post-incubation.

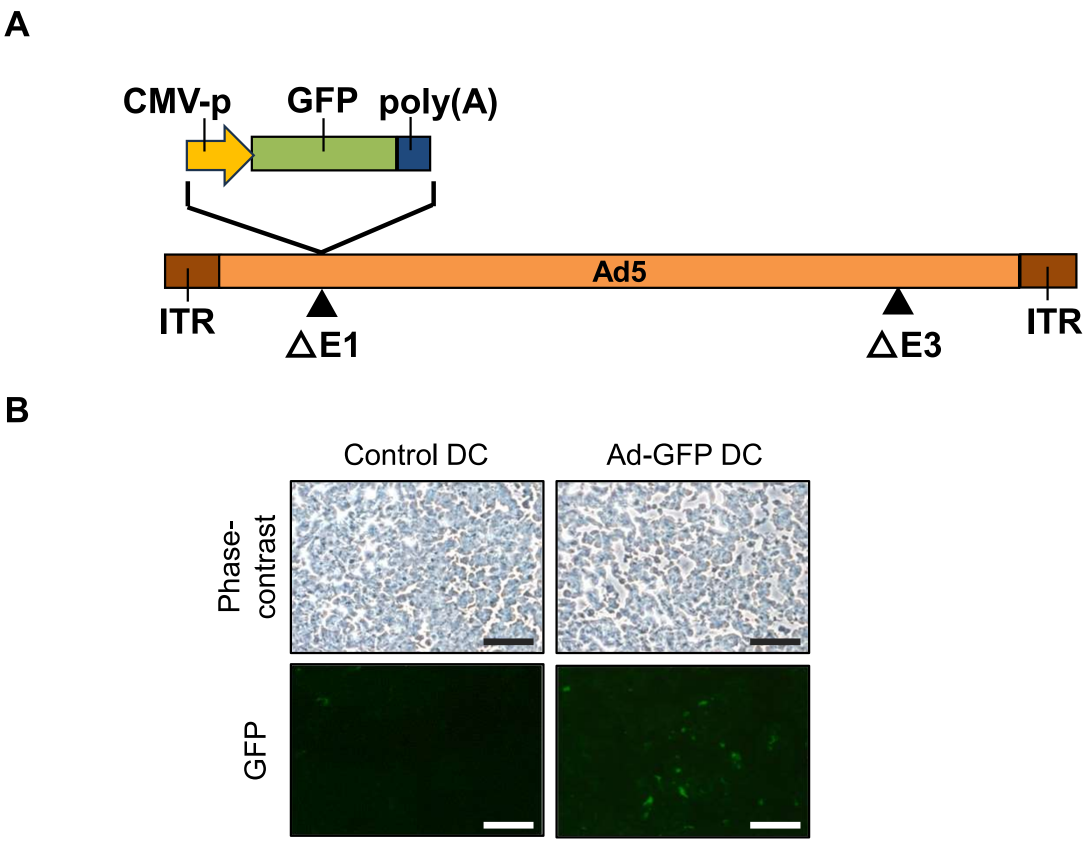

Supplementary Figure 4. Evaluation of GFP+ cells at inguinal lymph nodes of Ad-GFP DC-treated mice. **A**, Schematic structure of the replication-deficient adenovirus Ad-GFP, in which the CMV promoter drives expression of the *GFP* gene. **B**, Representative photographs of inguinal lymph nodes resected from BALB/c mice treated with control DCs or Ad-GFP-DCs. Scale bars, 50 µm. Ad5: Adenovirus serotype 5; CMV: cytomegalovirus; GFP: green fluorescent protein; ITR: inverted terminal repeat.

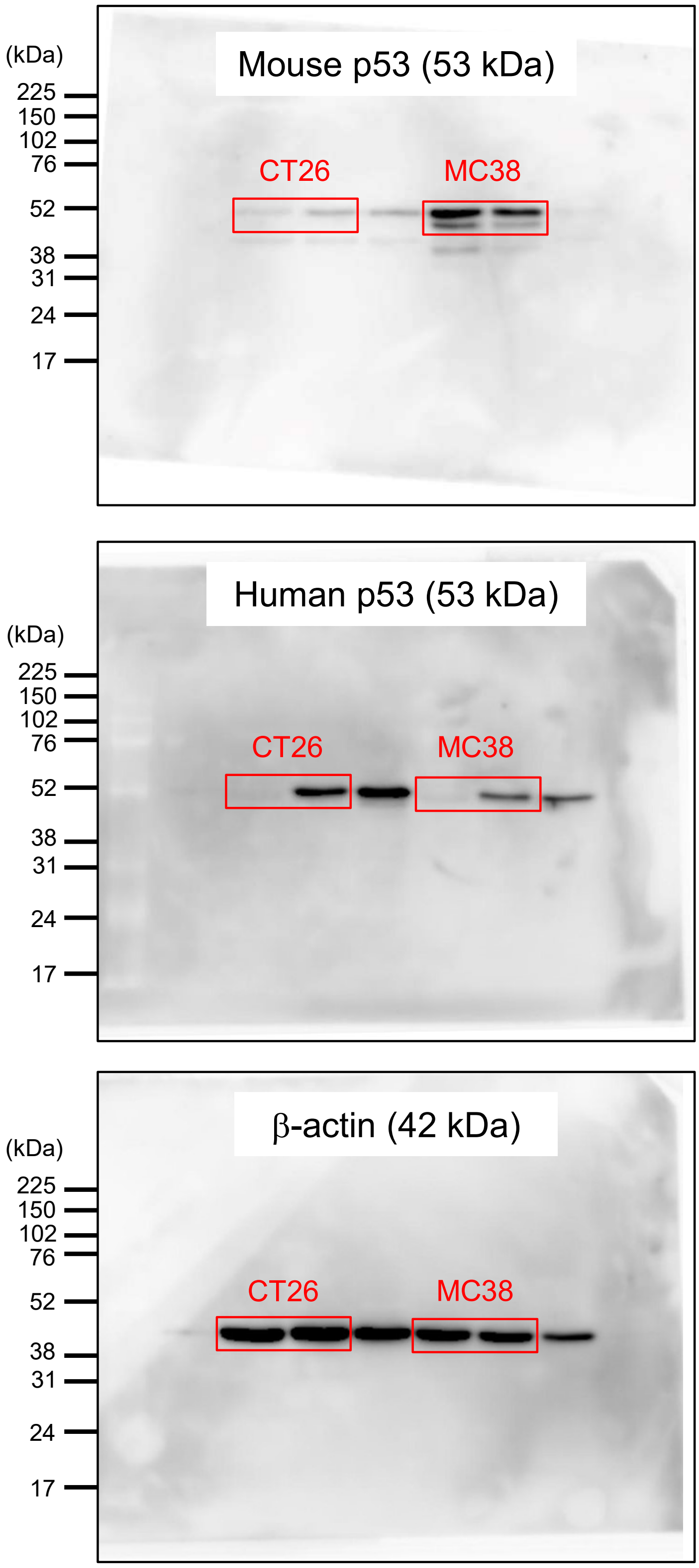

Supplementary Figure 5. Full images of Figs. 2C and 6C.

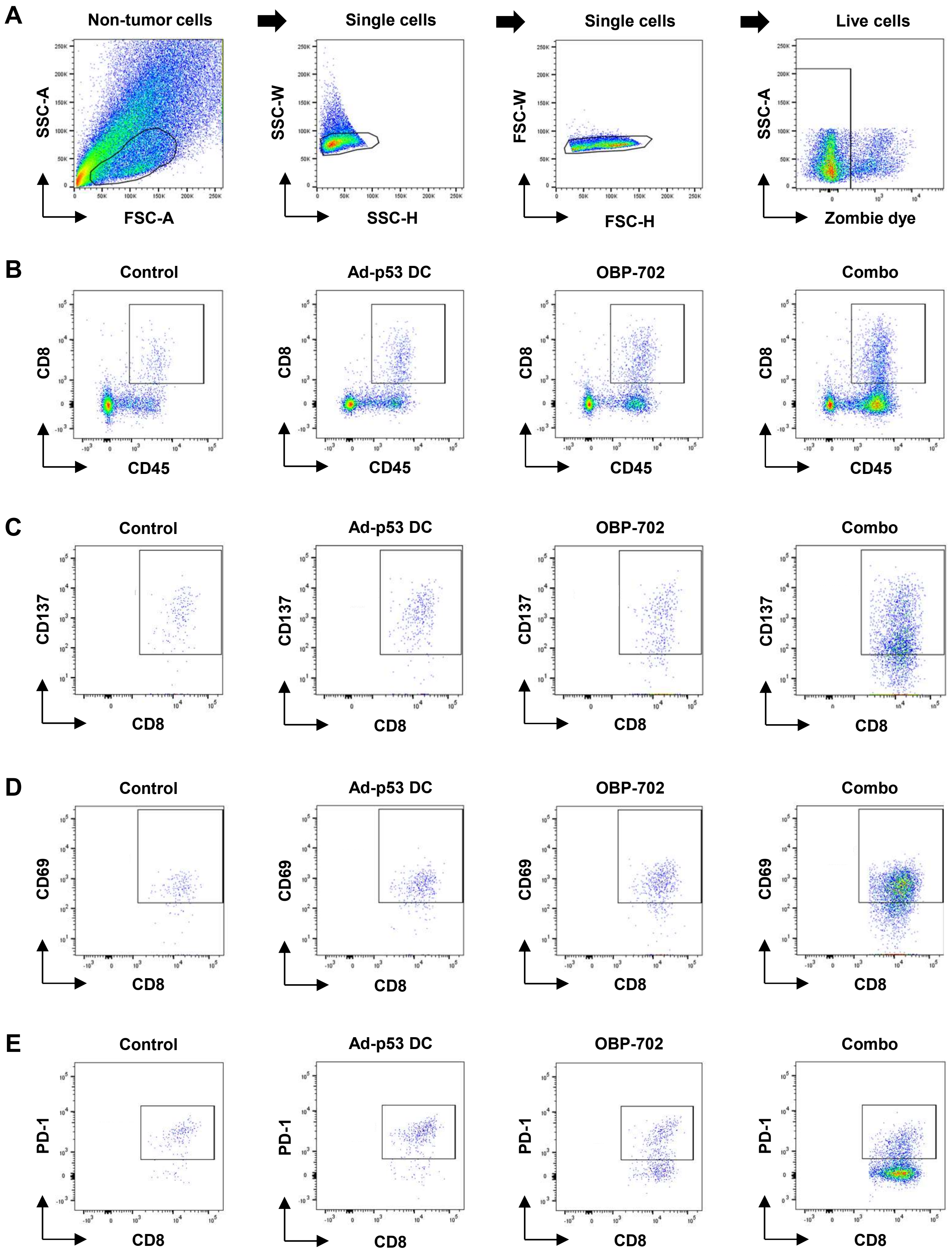

Supplementary Figure 6. Gating strategy for CD8+ T cells in FACS analysis. A, Representative FSC, SSC, and zombie dye plots with gating for non-tumor cells, single cells, and live cells. **B**, Representative CD45 and CD8 plots with gating for CTLs in each group. **C**, **D**, **E**, Representative CD8 and CD137 (**C**), CD69 (**D**), and PD-1 (**E**) plots with gating for CTLs in each group.

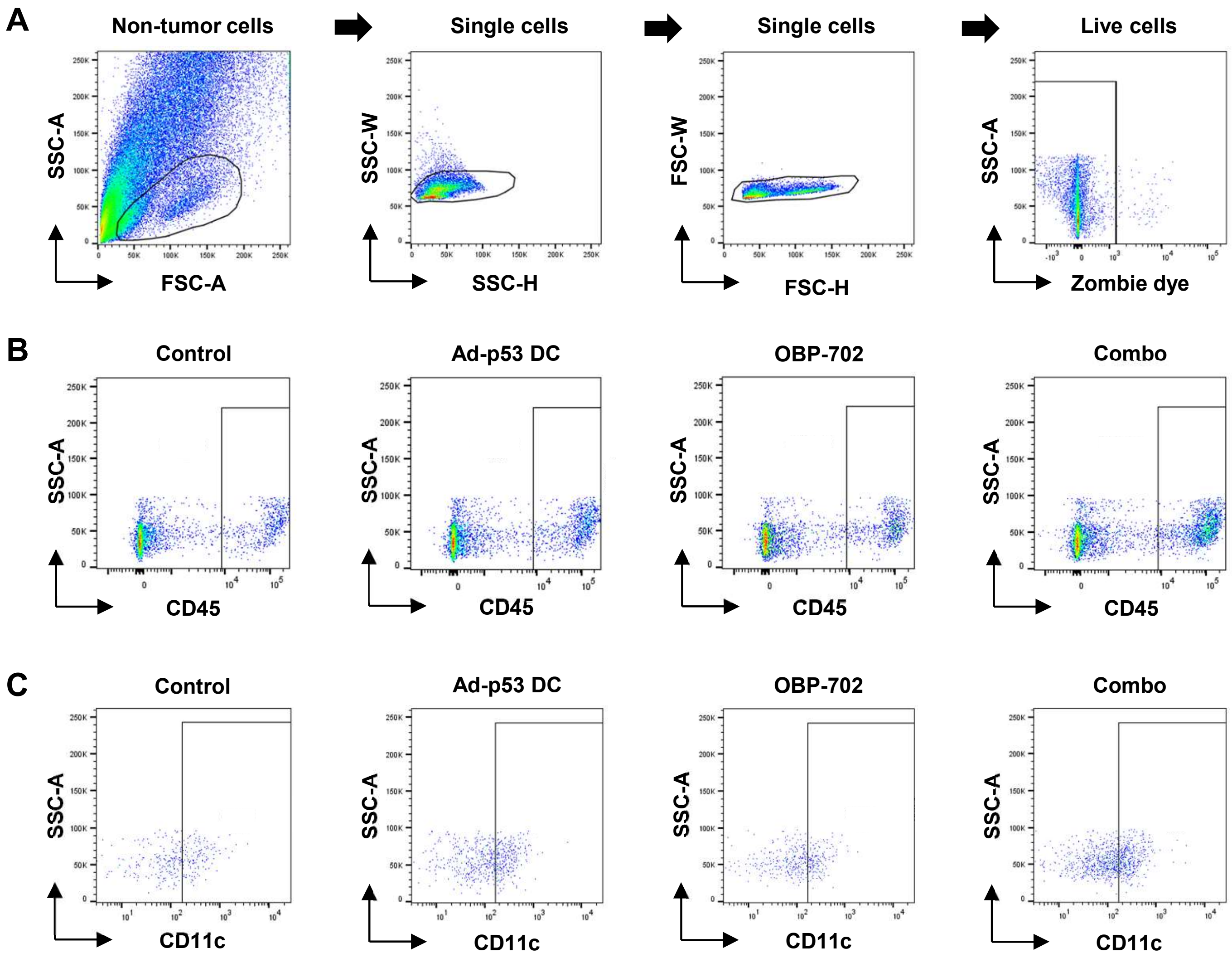

Supplementary Figure 7. Gating strategy for CD11c+ DCs in FACS analysis. A, Representative FSC, SSC, and zombie dye plots with gating for non-tumor cells, single cells, and live cells. **B**, Representative CD45 plots with gating for hematopoietic cells in each group. **C**, Representative CD11c plots with gating for DCs in each group.

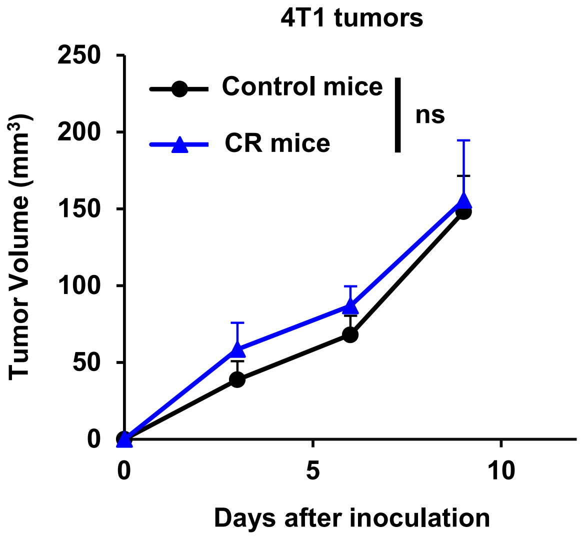

Supplementary Figure S8. Comparable growth of 4T1 tumors between control and CR mice. Combination therapy-treated complete response (CR) mice and control mice were subcutaneously re-inoculated with CT26 cells and 4T1 murine breast cancer cells. Data are expressed as mean ± SD (n = 3). Statistical significance was determined using the Student t test. ns, not significant.

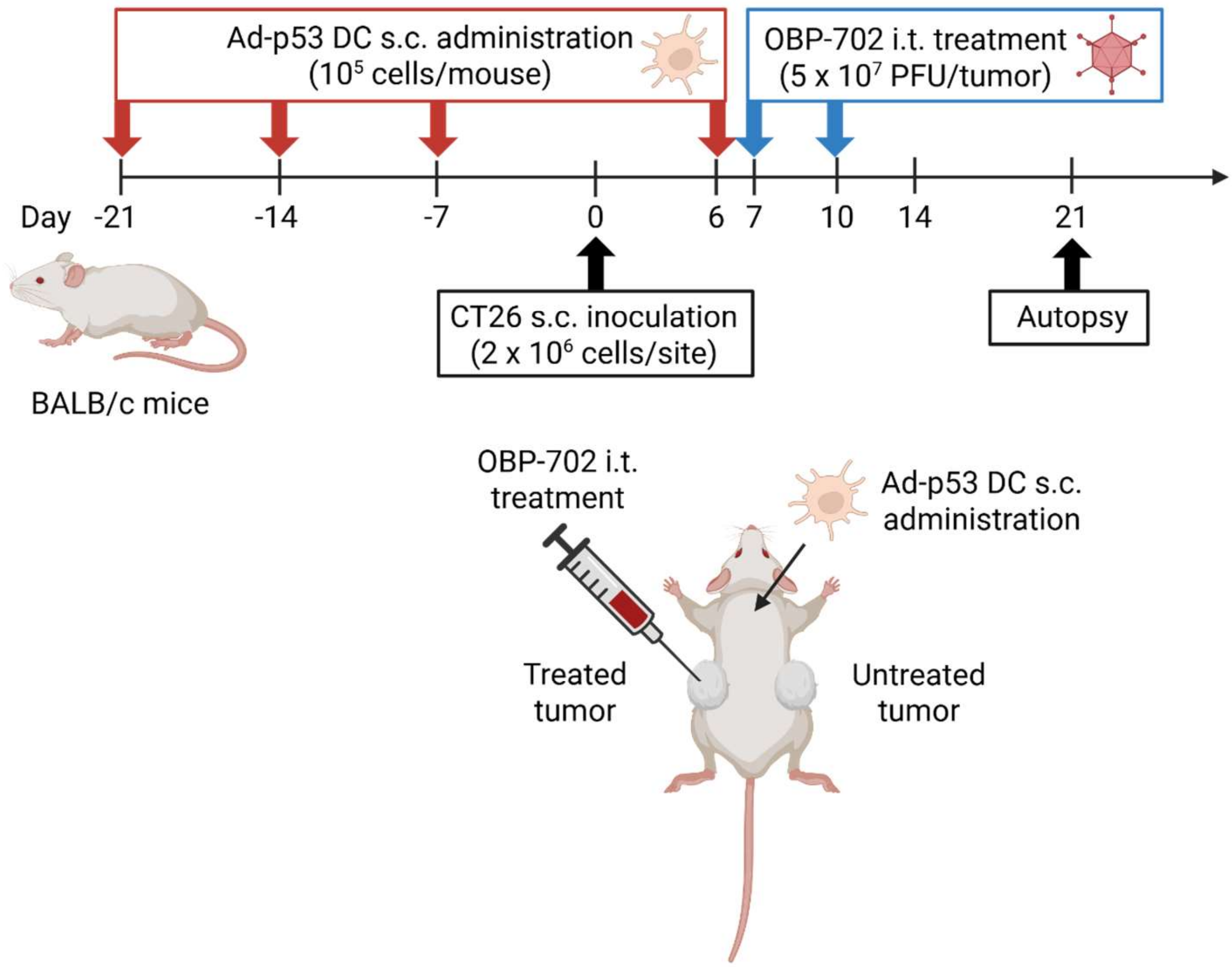

Supplementary Figure 9. Schematic illustration of the experimental protocol of bilateral CT26 tumor models. Figures were generated using BioRender.

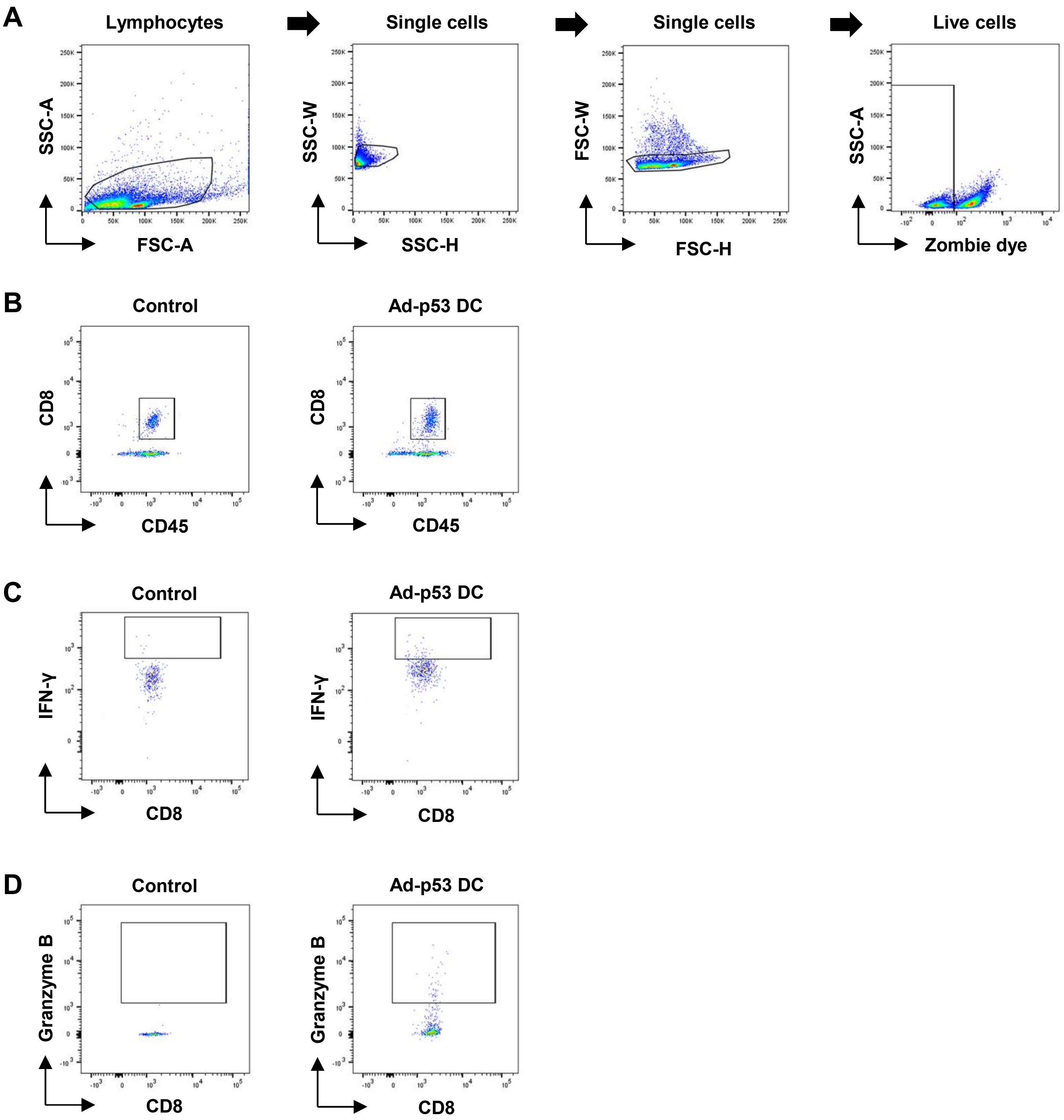

Supplementary Figure 10. Gating strategy for IFN-γ+ and granzyme B+ T cells in FACS analysis. A, Representative FSC, SSC, and zombie dye plots with gating for lymphocytes, single cells, and live cells. **B**, Representative CD45 and CD8 plots with gating for CTLs in each group. **C**, **D**, Representative CD8 and IFN-γ (**C**) and granzyme B (**D**) plots with gating for CTLs in each group.

Table S1. Identification of p53 peptides that were commonly expressed on the MHC molecules of Ad-p53 DCs and OBP-702-infected tumor cells. Candidate epitopes highlighted using font were commonly expressed on MHC-II molecules of Ad-p53 DCs and MHC-I molecules of OBP-702-infected tumor cells.

Cell	Treatment	MHC type	Peptide	Human p53 region	
				Start position	End position
DC	Control	MHC-II	FLHSGTAKSVTCTYSPALNKMF	113	134
DC	Control	MHC-II	HSGTAKSVTCTYSPALNKMFC	115	135
DC	Control	MHC-II	LAKTCPVQLWVDSTPPPGTRVRAMA	137	161
DC	Control	MHC-II	MNRRPILTIITLEDS	246	260
DC	Control	MHC-II	RGRERFEMFRELNEALELKD	333	352
DC	Control	MHC-II	RERFEMFRELN	335	345
DC	DL312	MHC-II	AAPAPAPSWPLSSSVPSQK	83	101
DC	DL312	MHC-II	KTYQGSYGFRLGFL	101	114
DC	DL312	MHC-II	TCTYSPALNKMFCQLAKTCPVQL	123	145
DC	DL312	MHC-II	PALNKMFCQLAKTCPV	128	143
DC	DL312	MHC-II	IYKQSQHM	162	169
DC	DL312	MHC-II	DGLAPPQHLIRVE	186	198
DC	DL312	MHC-II	EVGSDCTTIHYNYMCNSSCMGGMNRRP	224	250
DC	DL312	MHC-II	GEYFTLQIRGRERFEM	325	340
DC	DL312	MHC-II	YFTLQIRGRERFEMF	327	341
DC	DL312	MHC-II	GRERFEMFRELNEALELK	334	351
DC	DL312	MHC-II	ALELKDAQAGKEP	347	359
DC	Ad-p53	MHC-II	GPDE**APRMPEAAPPVAPAPAA**PTP	59	82
DC	Ad-p53	MHC-II	GPDE**APRMPEAAPPVAPAPAA**PTPAAP	59	85
DC	Ad-p53	MHC-II	SSVPSQKTYQGSYGFRLGFLHSGTAK	95	120
DC	Ad-p53	MHC-II	HSGTAKSVTCTYSPALNKMFCQL	115	137
DC	Ad-p53	MHC-II	LAKTCPVQLWVDSTPPPG	137	154
DC	Ad-p53	MHC-II	QSQHMTEVVRR	165	175
DC	Ad-p53	MHC-II	HYNYMCNSSCMGGMNRR	233	249
DC	Ad-p53	MHC-II	NLLGRNSFEVRVCACPG	263	279
CT26	Control	MHC-I	SQKTYQGSYGFRLG	99	112
CT26	Control	MHC-I	LNKMFCQLAKTCPVQLWV	130	147
CT26	Control	MHC-I	KMFCQLAKTCPVQL	132	145
CT26	Control	MHC-I	NSFEVRVCACPGRDRRTEEE	268	287
CT26	Control	MHC-I	QAGKEPGGSRA	354	364
CT26	OBP-301	MHC-I	APAPSWPLSSSVPSQKTYQG	86	105
CT26	OBP-301	MHC-I	TAKSVTCTYSPALNKMFCQLAK	118	139
CT26	OBP-301	MHC-I	DCTTIHYNYMCNSSCMGGMNRRPIL	228	252
CT26	OBP-301	MHC-I	HHELPPGSTKRA	296	307
CT26	OBP-301	MHC-I	KKPLDGEYFTLQIRGRERFEMF	320	341
CT26	OBP-702	MHC-I	**APRMPEAAPPVAPAPAA**PTPAAPAPAPSWP	63	92
CT26	OBP-702	MHC-I	HSGTAKSVTCTYSPALNKM	115	133
CT26	OBP-702	MHC-I	KTCPVQLWVDSTPPPGTRVRAMAIYK	139	164
CT26	OBP-702	MHC-I	PPPGTRVRAMAIYKQSQHMTEVV	151	173
CT26	OBP-702	MHC-I	RCSDSDGLAPPQHLIRVE	181	198
CT26	OBP-702	MHC-I	APPQHLIRVEGNLRVEY	189	205

CT26	OBP-702	MHC-I	TIHYNYMCNSSCM	231	243
CT26	OBP-702	MHC-I	IHYNYMCNSSCMGGMNR	232	248
CT26	OBP-702	MHC-I	CNSSCMGGMNR	238	248
MC38	Control	MHC-I	SWPLSSSVPSQKTYQGSY	90	107
MC38	Control	MHC-I	HLIRVEGNLRVEYLD	193	207
MC38	Control	MHC-I	DRNTFRHSVVVP	208	219
MC38	Control	MHC-I	EPHHELPPGSTKRA	294	307
MC38	Control	MHC-I	KDAQAGKEPGGSRA	351	364
MC38	OBP-301	MHC-I	DGLAPPQHLIRVE	186	198
MC38	OBP-301	MHC-I	GEPHHELPPGSTKRALPNN	293	311
MC38	OBP-301	MHC-I	RALPNNTSSSPQP	306	318
MC38	OBP-702	MHC-I	DLWKLLPENNVLSPLPSQAMDDLMLSPDDI	21	50
MC38	OBP-702	MHC-I	ENNVLSPLPSQAMDDLML	28	45
MC38	OBP-702	MHC-I	NVLSPLPSQAMDDL	30	43
MC38	OBP-702	MHC-I	PDDIEQWFTEDPGPDE**APRMPE**	47	68
MC38	OBP-702	MHC-I	DE**APRMPEAAPPVAP**	61	75
MC38	OBP-702	MHC-I	MTEVVRRCPHHE	169	180
MC38	OBP-702	MHC-I	YMCNSSCMGGMNRRPILT	236	253
MC38	OBP-702	MHC-I	PGRDRRTEEENLRKKGEPHH	278	297
MC38	OBP-702	MHC-I	ENLRKKGEPHHELPPGST	287	304
MC38	OBP-702	MHC-I	NEALELKDAQAGKEPGGSR	345	363
MC38	OBP-702	MHC-I	KDAQAGKEPGGSRA	351	364

MHC-I, major histocompatibility complex class I

MHC-II, major histocompatibility complex class II

Supplementary Table 2. Primary antibodies used for flow cytometry

Molecule	Clone	Fluorochrome	Source	Identifier	Dilution
Mouse CD8a	53-6.7	APC	BioLegend	100711	1:100
Mouse CD11c	N418	PerCP	BioLegend	117316	1:100
Mouse CD11c	N418	PE	BioLegend	117307	1:50
Mouse CD45	30-F11	PerCP	BioLegend	103129	1:100
Mouse CD69	H1.2F3	BV510	BioLegend	104531	1:50
Mouse CD86	GL-1	FITC	BioLegend	105005	1:50
Mouse CD103	2E7.	Alexa-647	BioLegend	121409	1:50
Mouse CD137	17B5	PE	BioLegend	106105	1:100
Mouse CCR7	4B12	BV421	BioLegend	120119	1:100
Mouse Granzyme B	QA16A02	PE	BioLegend	372207	1:50
Mouse Interferon-gamma	XMG1.2	APC-Cy7	BioLegend	505849	1:100
Mouse MHC class I	M1/42	FITC	BioLegend	125507	1:50
Mouse MHC class II	M5/114.15.2	PE-Cy7	BioLegend	107629	1:50
Mouse PD-1 (CD279)	29F.1A12	FITC	BioLegend	135213	1:40
Propidium Iodide			BioLegend	421301	
Zombie Aqua			BioLegend	423101	1:1000
Zombie NIR			BioLegend	423105	1:1000
